# New Mechanisms and Therapeutic Targets in Systemic Lupus Erythematosus

**DOI:** 10.1002/mco2.70246

**Published:** 2025-06-09

**Authors:** Jingru Tian, Hang Zhou, Wei Li, Xu Yao, Qianjin Lu

**Affiliations:** ^1^ Hospital for Skin Diseases Institute of Dermatology Chinese Academy of Medical Sciences and Peking Union Medical College Nanjing China; ^2^ Key Laboratory of Basic and Translational Research on Immune‐Mediated Skin Diseases Chinese Academy of Medical Sciences Nanjing China; ^3^ Jiangsu Key Laboratory of Molecular Biology for Skin Diseases and STIs Nanjing China; ^4^ Department of Allergy and Rheumatology Hospital for Skin Diseases Institute of Dermatology Chinese Academy of Medical Sciences and Peking Union Medical College Nanjing China; ^5^ Department of Dermatology Huashan Hospital Fudan University Shanghai China

**Keywords:** epidemiology, mechanism, systemic lupus erythematosus, therapeutic targets, type I interferon

## Abstract

Systemic lupus erythematosus (SLE) is a multifaceted dautoimmune disease driven by complex interactions among genetic, environmental, and sex‐related factors. Central to its pathogenesis are type I interferons (IFN‐I) and autoantibodies that target nucleic acids and nucleic acid‐binding proteins. These mediators, often triggered by environmental stimuli in genetically susceptible individuals, promote sustained immune activation and chronic inflammation. Despite advances in understanding the immunological landscape of SLE, the precise initiating triggers and early molecular events remain incompletely defined. Recent studies have highlighted the destabilization of innate immune cells, particularly dendritic cells and monocytes, as critical early events in the pathogenesis of SLE. These alterations precede and potentially initiate the downstream activation of autoreactive lymphocytes. This review provides an updated synthesis of key epidemiological findings, emerging pathogenic mechanisms, potential therapeutic targets, and advances in translational and clinical research. Particular attention is given to recent insights into disease triggers and early pathological processes, especially the destabilization of innate immune cells. By consolidating these advances, this review aims to refine our understanding of the early immune dysregulation in SLE and to support the development of more precise, mechanism‐based therapeutic strategies.

## Graphical abstract

Systemic lupus erythematosus (SLE) is a multiple organ involved autoimmune disorder with heterogeneous clinical manifestations. Despite hallmark features such as elevated type I interferon and the production of numerous autoantibodies, the underlying pathogenic mechanisms remain elusive. In this review, we systematically summarize the emerging pathogenic mechanisms in SLE and explore the advances in identifying potential therapeutic targets that could benefit the management of this challenging disease.

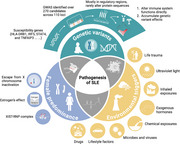

## 1 Introduction

Systemic lupus erythematosus (SLE) is a complex autoimmune disorder that affects multiple organs and is characterized by the aberrant production of type I interferons (IFN‐I) and autoantibodies and by the deposition of immune complexes (ICs). The disease is highly heterogeneous, presenting with a broad spectrum of clinical manifestations, which complicates diagnosis, particularly in its early stages. The chronic and relapsing‐remitting nature of SLE often necessitates prolonged corticosteroid therapy, which, while managing disease activity, contributes to cumulative damage in classic target organs and tissues. This progressive tissue injury can lead to severe comorbidities and, in advanced cases, mortality.

Over the past few decades, the understanding of the pathogenesis of SLE has focused primarily on immune cells, particularly aberrant T and B lymphocytes. This complex cell interplay has led to extensive exploration of the proportions and functions of abnormal subsets of T cells and B cells, further promoting translational research on lymphocytes. However, adaptive immunity, represented by T and B lymphocytes, generally resides in the downstream phase of immune responses. Lupus is an autoimmune disease that primarily affects young to middle‐aged women, yet the epigenetic changes induced by environmental factors and hormones, as well as age‐ and sex‐related triggers, remain largely unknown, as do the early pathological processes of the disease.

Thus, this review aims to provide a comprehensive synthesis of recent advances in epidemiology, pathogenesis, therapeutic targets, and translational research related to SLE. By reviewing the current landscape of SLE research, we seek to identify major factors that contribute to the pathogenesis of the disease. Particular emphasis is placed on the environmental and genetic triggers of SLE, as well as the destabilization of innate immune cells, offering insights into the early pathological events that drive disease onset and progression.

## 2 Lupus Epidemiology

SLE can manifest at any age and in individuals of any gender, though it predominantly affects women of reproductive age [1]. Existing research is often confined to localized regions, with most developing countries lacking epidemiological data on SLE [2]. Overall, the worldwide rate of new SLE cases is calculated at 5.14 for every 100,000 individuals per year, with around 400,000 new cases each year. The global prevalence is estimated at 43.7 per 100,000 people, affecting approximately 3.41 million individuals annually, with a female‐to‐male ratio of about 9:1 [2]. However, these estimates rely on limited, non‐large‐scale studies, and significant discrepancies exist even among multiple studies conducted within the same country (Figures S1 and S2). Despite attempts to balance data variances, prediction bias remains a challenge.

Overall, SLE disproportionately affects adults, women, and individuals residing in high‐income countries or regions [2]. However, with few exceptions, mortality rates among SLE patients in low‐ and middle‐income countries/regions are significantly higher than those in high‐income countries/regions [3, 4, 5]. These differences linked to economic and population characteristics may be credited to more advanced healthcare systems, better availability to specialists, improved insurance documentation, and elevated public consciousness in high‐income countries/regions [2, 3]. Besides, ethnic and racial diversity significantly influence prevalence and incidence. Studies consistently show higher SLE rates among Asian, Black, Hispanic, and Indigenous groups [6, 7, 8, 9]. For example, in the United States, American Indian/Alaska Native women have the highest prevalence (270.6 per 100,000 people), followed by Black women (230.9). Incidence is highest in Black women (15.9 per 100,000 person‐years) and American Indian/Alaska Native women (10.4), all exceeding rates in White women (5.7) [9, 10].

Regarding disease outcomes, African Americans tend to experience earlier disease onset and increased disease severity, even after controlling for socioeconomic factors. Among African Americans, 40.5% of patients develop nephropathy, and 15.3% progress to end‐stage renal failure, compared with 18.8 and 4.5%, respectively, among White patients [11]. Data from Asian countries similarly indicate a higher risk of SLE‐related complications among local ethnic groups, with severe kidney involvement being particularly prevalent [12]. Apart from renal involvement, a recent meta‐analysis found that the age of SLE onset is associated with the symptoms and incidence of neuropsychiatric SLE (NPSLE). The cumulative incidence of NPSLE in the early‐onset group (under 50 years old) was approximately 1.41 times higher than that in the late‐onset group (diagnosed at or after 50 years old). The incidence of seizures and psychosis in early‐onset SLE patients were 1.68 and 1.72 times higher, respectively, than those in late‐onset patients. In contrast, the incidence of peripheral neuropathy showed the opposite trend, with the early‐onset group having only 0.64 times the rate of the late‐onset group [13].

## 3 Pathogenesis of Disease

**FIGURE 1:**
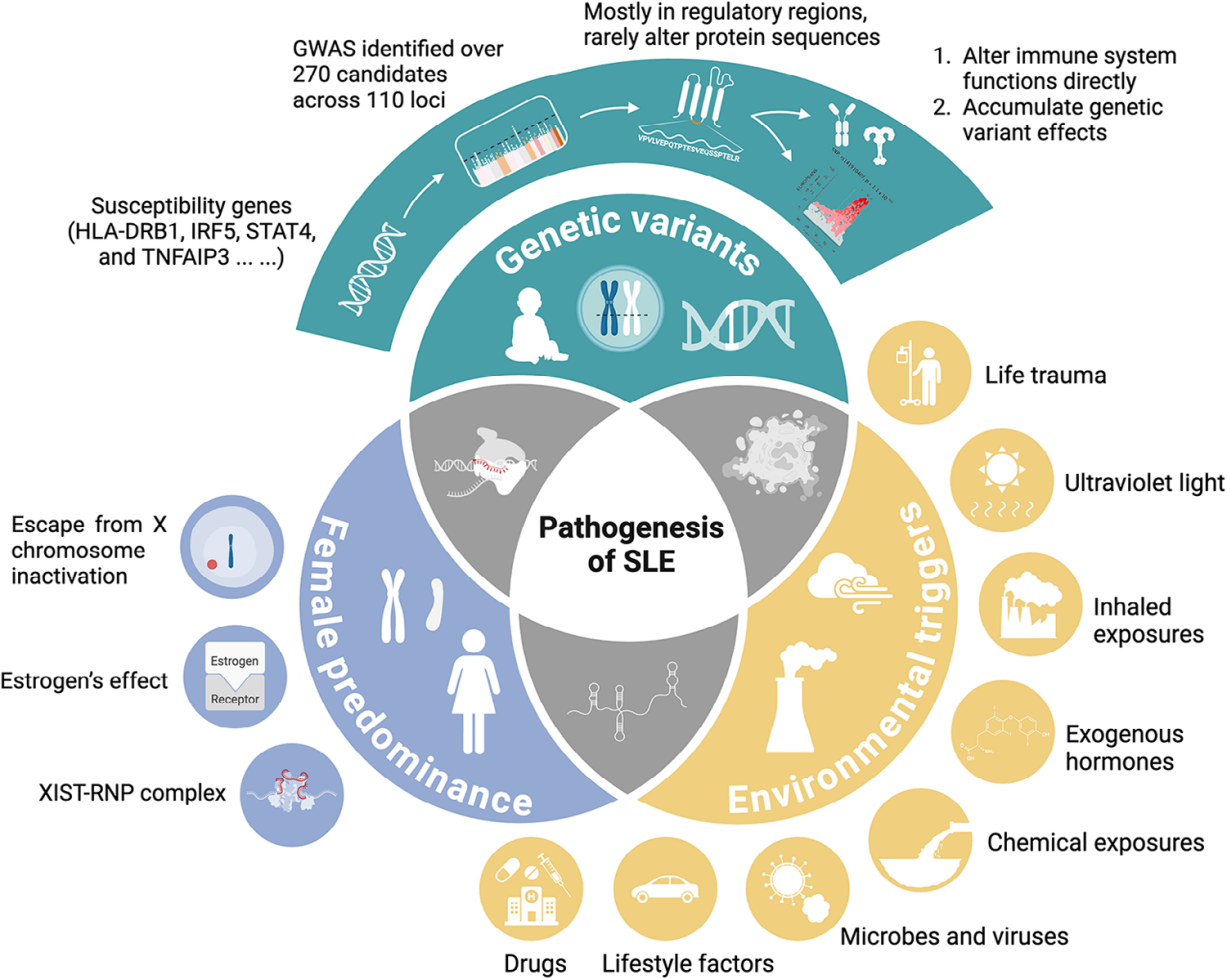
A broad view of the pathogenesis of SLE. The pathogenesis of SLE arises from the interplay of genetic susceptibility, female predominance, and environmental triggers. Most genetic risk variants reside in regulatory regions and collectively disrupt immune regulation, particularly in pathways involving type I interferons, NF‐κB signaling, and lymphocyte activation. Female‐biased incidence is linked to X chromosome inactivation escape, estrogen‐mediated immune enhancement, and the formation of immunogenic XIST–RNP complexes. Environmental factors such as UV radiation, microbial dysbiosis, and chemical exposures further amplify disease risk. These diverse triggers converge on common downstream immune pathways, including IFN‐I signaling, autoreactive T and B cell activation, and persistent autoantibody production, driving the breakdown of self‐tolerance and multiorgan inflammation. GWAS, genome‐wide association study; HLA‐DRB1, human leukocyte antigen DR beta 1; IFN‐I, type I interferons; IRF5, interferon regulatory factor 5; STAT4, signal transducer and activator of transcription 4; TNFAIP3, tumor necrosis factor alpha‐induced protein 3; XIST–RNP, X‐inactive specific transcript‐ribonucleoprotein.

SLE and the diverse types of cutaneous lupus erythematosus together constitute the lupus disease spectrum. However, as our understanding of lupus increases, the traditional perception of this spectrum is being challenged. There is even growing debate over whether this SLE itself, characterized by unknown pathogenesis, diverse clinical manifestations, a lack of diagnostic biomarkers, and complex cellular and molecular changes, might actually be a collection of distinct diseases with similar symptoms [14]. The limited success of current translational research has led researchers to reassess this ancient and complex disease. Despite the two hallmark features of SLE, namely, excessive production of IFN‐I and characteristic autoantibody generation, targeting plasmacytoid dendritic cell (pDC)‐derived IFN‐I in phase 2 trials did not yield significant changes in disease activity endpoints for SLE [15]. Treatments targeting B cells, which are the source of these antibodies, achieved Systemic Lupus Erythematosus Responder Index scores of 51–58% and efficacy renal response rates of 43% in phase 3 multinational, multicenter trials for active SLE and lupus nephritis (LN), compared with 44 and 32% in the placebo groups, respectively [16, 17]. These clinical outcomes indicate that although patients exhibit similar systemic damage and pathological features, the initial triggering events and upstream pathological processes may be heterogeneous. These early responses are likely masked by the amplified downstream immune response during disease progression. Accordingly, the pathological processes from the onset of SLE to the production of characteristic IFN‐I and the breakdown of self‐tolerance are reviewed here, aiming to identify commonalities within this heterogeneity (Figure 1).

### 3.1 Common Genetic Variants

As early as 1987, Eisenberg and colleagues [18] reported that only 25% of 5‐month‐old autoimmune MRL/Mp‐lpr/lpr mice produced anti‐Smith (Sm) autoantibodies and that the production of anti‐Sm was independent of genetics, the rearing environment, or parental influences. This finding suggests that while the risk of autoimmunity is genetically controlled, it is also influenced by individual stochastic factors. SLE is widely accepted as a polygenic disorder influenced by environmental and hormonal factors.

The genetic background of SLE has long been a focus of research, and several key susceptibility genes have been identified, including HLA‐DRB1, IRF5, STAT4, and TNFAIP3, which are involved in immune regulation and exhibit increased variant frequencies in SLE patients [19, 20, 21]. However, owing to the long duration and complexity of in vivo studies, only a limited number of single‐gene mutations have been linked to the onset of SLE, and animal models based on these findings are lacking.

There is evidence of familial aggregation in SLE patients. An earlier twin registry study demonstrated that 24% of monozygotic twins and 2% of dizygotic twins were concordant for the disease [22]. A large‐scale study based on Taiwan's national health insurance records suggested that within families with an SLE patient, the individual risk for developing SLE and other autoimmune diseases is elevated. Specifically, the risk of developing SLE was elevated by 315.94 times for twins of patients with SLE, 23.68 for siblings, 14.42 for offspring, and 4.44 for spouses who do not share genetic similarities [23]. In the same study, the phenotypic variation observed in SLE patients was estimated to be attributable to 43.9% genetic heritability, 25.8% shared environmental factors, and 30.3% nonshared environmental influences [23]. Overall, an environmentally regulated polygenic additive model is considered the most suitable framework for SLE [24].

In recent years, several extensive genome‐wide association studies have revealed more than 270 candidate disease genes across 110 loci in SLE patients, including regions within the human leukocyte antigen (HLA) locus [25]. Many of these variants are directly involved in immune system functions, particularly the IFN‐I pathway, the NF‐κB pathway, and T‐cell and B‐cell activation and differentiation [19, 20, 26, 27]. Most genetic variants associated with SLE are found in regulatory regions rather than coding regions [25, 26, 27, 28, 29, 30]. Only a small number of risk variants result in changes to protein sequences, as exemplified by Fc receptor variants [31]. However, these variants alone likely do not significantly contribute to the pathogenesis of SLE unless they are combined with mutations in certain regulatory regions or other genes involved in maintaining immune tolerance [32]. The impact of genetic variation is both nonlinear and cumulative, with certain alleles exerting greater influence as the overall genetic risk burden increases [33]. Specific genetic variations associated with SLE can be found in recent review articles [14, 34].

### 3.2 Female Predominance

The mechanisms underlying the extreme sex disparity in SLE remain unclear. In addition to well‐known factors such as escape from X chromosome inactivation and estrogen's effect on lymphocyte activation [35], recent findings by Dou and colleagues [36] revealed that the long noncoding RNA Xist, which regulates the random inactivation of one X chromosome in females, interacts with various RNA‐binding proteins to form Xist–RNPs. This complex includes multiple self‐antigen components that contribute to autoimmunity. Since Xist is expressed only in female mammalian cells, it drives the sex‐biased nature of autoimmune diseases [36].

### 3.3 Environmental Triggers

In addition to genetic factors, environmental factors are significant contributors to the onset and progression of SLE. Common candidate environmental triggers include ultraviolet (UV) light, microbes and viruses, exposure to inhaled substances, other chemical exposures, exogenous hormones, lifestyle factors, life trauma, and drugs.

#### 3.3.1 Microbes

Recent studies have revealed associations between microbial imbalances within the gut, dermis, oral cavity, vaginal cavity, bladder and plasma, and SLE pathogenesis [37, 38]. Among these, research on the gut microbiota has been the most extensive, and researchers have established a link between the gut microbiota and SLE. Compared with healthy controls, patients with SLE generally exhibit a reduced Firmicutes/Bacteroidetes (F/B) ratio and lower alpha diversity [39]. Specific gut bacteria, such as Bacilli, Lactobacillales, and *Ruminococcus gnavus*, are related to increased SLE risk and disease activity, whereas beneficial microbes such as Bifidobacterium may have protective effects [40]. The fungal microbiota in patients with SLE also shows reduced diversity, with certain fungi being more prevalent in patients with SLE [41]. Microbial shifts are observed in both human studies and lupus‐prone animal models, where altered gut microbiota patterns are linked to immune responses and disease severity [42].

The gut microbiome plays a crucial role in SLE pathogenesis via various pathways, including intestinal permeability, molecular mimicry, immune system dysfunction, and epigenetic modifications. Disrupted gut barriers in SLE allow bacterial translocation, as evidenced by *Lactobacillus reuteri*, which is found in internal organs of SLE mouse models, whereas elevated gut barrier markers such as fecal albumin in patients further link increased intestinal permeability to disease progression [43, 44, 45]. Additionally, molecular mimicry, where microbial antigens resemble self‐antigens, triggers autoimmunity. For example, in germ‐free mice, *Bacteroides thetaiotaomicron* induces immune responses against Ro60, leading to kidney immune complexes (ICs) deposition, and peptides from *Akkermansia muciniphila* mimic SLE‐related antigens, potentially activating T and B cells [46, 47]. Moreover, specific gut microbes, such as *Ruminococcus gnavus*, are associated with immune dysregulation in SLE, which is characterized by increased Th17 activation and altered Firmicutes/Bacteroidetes ratios [48, 49]. In line with these findings, studies in ZAP‐70‐mutated mice have shown that altered gut microbiota promote Th17 differentiation and systemic autoimmunity [48]. Furthermore, gut metabolites such as short‐chain fatty acids (SCFAs) influence immune function and gene expression, thereby affecting SLE progression [50, 51]. The gut bacteria‐influenced tryptophan metabolism plays a role in the severity of SLE, with high dietary tryptophan exacerbating symptoms in mice, and fecal transplants from these mice induce autoimmune phenotypes in germ‐free mice, underscoring the microbiome's role in disease development [52, 53]. Finally, the interplay between genetics and the microbiome is evident in studies where specific gut microbes influence autoimmune symptoms in mouse models, emphasizing the combined role of genetic and microbial factors in SLE pathogenesis [54, 55].

#### 3.3.2 UV Light

As the first line of defense against environmental stimuli, the local dermal immune microenvironment is composed of keratinocytes, immune cells, and immune molecules, which work together to maintain tissue homeostasis [56, 57]. Up to 92% of SLE patients exhibit photosensitivity, where exposure to sunlight or UV radiation triggers or exacerbates the disease [58, 59]. Compared with other tissues or immune cells in the body, keratinocytes, which form the skin barrier and encounter environmental stimuli directly, are not only particularly numerous but also highly immunoreactive [60]. Studies have shown that keratinocytes in SLE patients are more sensitive to external stimuli and exhibit an enhanced inflammatory response than those in healthy individuals [61, 62, 63]. UV radiation can directly damage the DNA of keratinocytes, leading to the production of large amounts of free double‐stranded DNA (dsDNA), which serves as a “danger signal” [60, 64, 65]. These signals are recognized by cytoplasmic immune sensors such as cGAS, which then catalyze the formation of cGAMP [66]. cGAMP acts as a second messenger to activate the downstream STING pathway. The activation of STING further triggers the translocation of transcription factors such as IRF3 and NF‐κB to the nucleus, leading to the secretion of large amounts of IFN‐I, chemokines, and cytokines by keratinocytes and the subsequent activation of downstream immune cells [61‐63, 67, 68]. In addition, the upregulated IFN‐I in keratinocytes upon stimulation may also rely on the Toll‐like receptor (TLR)3 and RIG‐I (retinoic acid‐inducible gene I) pathways [69, 70].

The systemic effects of UV exposure remain to be fully elucidated. Skopelja‐Gardner and colleagues [71] reported that UV radiation not only stimulates neutrophil migration to the skin but also promotes neutrophil migration to the kidneys in an IL‐17A‐dependent manner, thereby triggering neutrophil‐dependent subclinical renal inflammation and injury. A single‐cell RNA sequencing (scRNA‐seq) study analyzing renal and skin lesion samples from SLE patients revealed that the transcriptomic score of IFN‐inducible genes in tubular cells was correlated with the chronic index, IgG deposition, and proteinuria. Additionally, IFN‐inducible genes were similarly upregulated in keratinocytes from skin areas without lesions and without exposure to sunlight in LN patients, suggesting a potential link between skin keratinocytes and renal involvement [72].

Overall, these findings suggest that UV‐induced skin dysfunction is closely associated with the onset and progression of SLE.

## 4 Altered Immunoregulation

**FIGURE 2:**
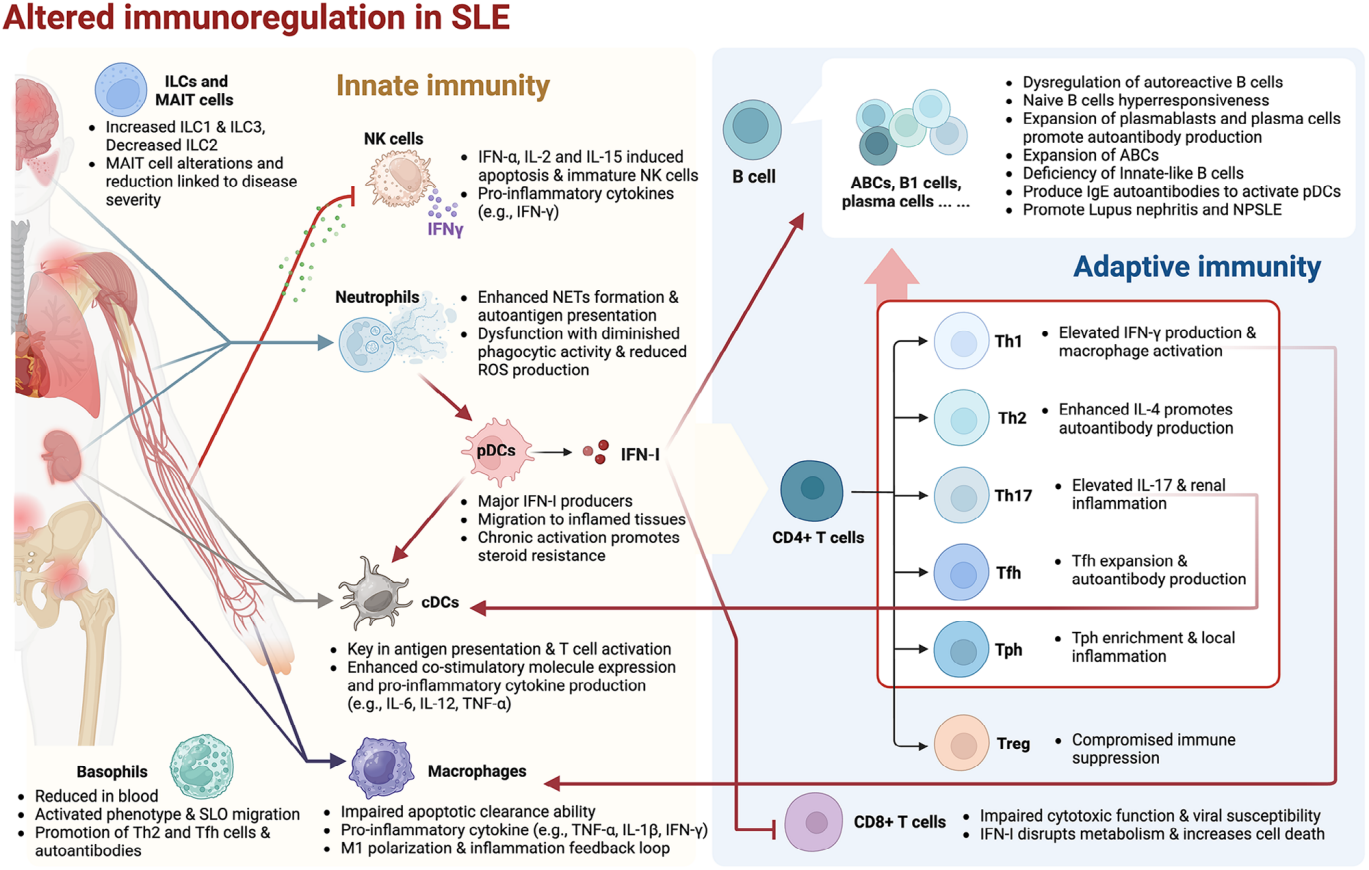
Disruption of immune homeostasis in SLE and the key changes and roles of specific immune cells. The pathogenesis of SLE is driven by a complex and interconnected network of innate and adaptive immune cells that collectively disrupt immune tolerance and sustain chronic inflammation. Neutrophils are among the earliest responders and promote disease progression through excessive formation of NETs, which not only expose nuclear autoantigens but also activate pDCs via TLR9, amplifying IFN‐I production. IFN‐I further activates both cDCs and macrophages, enhancing their capacity to present self‐antigens and perpetuate inflammatory cascades. Activated cDCs stimulate autoreactive CD4⁺ T cells, including Th1, Th17, Tfh, and Tph subsets, which in turn support B cell activation and pathogenic autoantibody production. Meanwhile, defective clearance of apoptotic cells by macrophages and skewing toward a proinflammatory M1 phenotype contribute to sustained antigen release and cytokine production, particularly TNF‐α, IL‐1β, and IFN‐γ. Basophils and NK cells, though fewer in number, amplify humoral responses and tissue inflammation through cytokine secretion and cell– interactions, particularly in lupus nephritis. Altered frequencies and functions of ILCs and MAIT cells have also been linked to tissue injury and immune dysregulation. On the adaptive side, loss of Treg function, metabolic exhaustion of CD8⁺ cytotoxic T cells, and expansion of age‐associated and extrafollicular B cells drive the breakdown of tolerance and persistent autoantibody production. Together, these immune cell populations form a dynamic and self‐reinforcing network that contributes to the systemic and organ‐specific manifestations of SLE. ABCs, antibody‐producing cells; B1 cell, B‐1 cell; cDCs, conventional dendritic cells; ILC, innate lymphoid cells; IFN‐α, interferon alpha; IFN‐γ, interferon gamma; IFN‐I, type I interferons; IL‐2, interleukin 2; IL‐4, interleukin 4; IL‐6, interleukin 6; IL‐12, interleukin 12; IL‐15, interleukin 15; IL‐17, interleukin 17; IL‐1β, interleukin 1 beta; MAIT, mucosal‐associated invariant T cells; NETs, neutrophil extracellular traps; NK cell, natural killer cell; NPSLE, neuropsychiatric systemic lupus erythematosus; pDCs, plasmacytoid dendritic cells; ROS, reactive oxygen species; SLO migration, secondary lymphoid organ migration; Th1, T helper 1; Th2, T helper 2; Th17, T helper 17; Tfh, T follicular helper; Tph, T peripheral helper; Treg, regulatory T cell; TNF‐α, tumor necrosis factor alpha; XIST–RNP, X‐inactive specific transcript‐ribonucleoprotein; IgE, immunoglobulin E.

Innate and adaptive immunity jointly drive the pathogenesis of SLE. The primary roles of immune cells and their pathological mechanisms in SLE are summarized in Figure 2.

### 4.1 Innate Immunity

Innate immune cells orchestrate a complex interplay of immune responses that drive SLE progression. Neutrophils, dendritic cells, macrophages, natural killer (NK) cells, basophils, mucosal‐associated invariant T (MAIT) cells, and innate lymphoid cells (ILCs) contribute to chronic inflammation and autoimmunity through mechanisms including the development of neutrophil extracellular traps (NETs), the production of IFN‐I, and the improper clearance of apoptotic cells. These cells not only initiate and amplify inflammatory pathways but also interact with adaptive immune cells, perpetuating the cycle of immune dysregulation that characterizes SLE.

#### 4.1.1 Neutrophils

Neutrophils promote SLE, particularly through their increased capacity to form NETs. NETs are networks of DNA and proinflammatory proteins that can entrap autoantigens such as chromatin anddsDNA, fueling the autoimmune response by presenting these autoantigens to the immune system [73, 74]. Neutrophils in SLE patients exhibit not only increased NET formation but also impaired clearance because of reduced serum DNase1 activity, which normally degrades NETs [75]. Inadequate NET clearance induces the activation of pDCs through TLR9, which results in the production of IFN‐I and exacerbates the disease [74]. Factors such as endothelin‐1 and hypoxia‐inducible factor‐1α drive the formation of NETs by inducing the expression of the stress‐response protein REDD1, promoting NETosis [76]. However, myeloid‐derived suppressor C‐type lectin‐like, an inhibitory C‐type lectin receptor, can directly recognize DNA within NETs and suppress NET formation and the development of a self‐inflammatory feedback loop through the reactive oxygen species (ROS)–PAD4 pathway [77]. Recent studies have shown that gasdermin D deficiency disrupts myeloid calcium influx, promoting granulopoiesis and worsening LN by enhancing NET formation [78]. Additionally, oxidized mitochondrial DNA can induce gasdermin D oligomerization, which is crucial for NET formation in SLE [79]. Furthermore, oxidized galectin‐1 in SLE patients fails to bind the inhibitory receptor VSTM1, leading to increased ROS levels in neutrophils, which in turn increase NET formation and inflammation [80].

NETs not only are implicated in tissue damage in SLE, as evidenced by their presence in discoid skin lesions and LN‐affected kidneys, but also help activate marginal zone B cells, promoting immunoglobulin class switching, somatic hypermutation, and antibody production [81]. Additionally, NET‐associated gene signatures have been identified in individuals with LN, linking neutrophil activity to specific clinical manifestations of SLE [82].

In addition to NET‐related mechanisms, neutrophils in SLE patients exhibit other dysfunctions that contribute to the disease. For example, they show diminished phagocytic activity and impaired ROS production, which are crucial for pathogen clearance and the regulation of apoptotic debris. A reduction in ROS production is often linked to genetic variants, such as those in NCF1, which impair the function of NADPH oxidase, a key enzyme in ROS generation [83, 84, 85]. A missense SNP in NCF1 has been associated with reduced ROS production and an increased IFN‐I signature, directly connecting neutrophil dysfunction with SLE pathogenesis [85, 86]. Moreover, glutathione peroxidase 4‐regulated ferroptosis in neutrophils has been shown to contribute to systemic autoimmunity [87].

In addition to these intrinsic neutrophil dysfunctions, external factors such as *Staphylococcus aureus* colonization in the skin can intensify inflammation, similar to that in SLE, via neutrophil activation through the IL‐23/IL‐17 axis, linking infection to autoimmunity [88]. The endoplasmic reticulum stress sensor IRE1α also amplifies neutrophil hyperactivity, further driving the autoimmune response in SLE [89].

#### 4.1.2 Dendritic Cells

DCs are pivotal antigen‐presenting cells (APCs) that serve as crucial links between innate and adaptive immune responses. In the context of SLE, both pDCs and conventional DCs (cDCs) play important roles in the initiation and perpetuation of autoimmune responses.

##### 4.1.2.1 Plasmacytoid Dendritic Cells

pDCs are a specialized subset of DCs that are major producers of IFN‐I, particularly IFN‐α, a key cytokine in SLE pathogenesis. pDCs express high levels of TLR7 and TLR9, which recognize single‐stranded RNA and unmethylated CpG DNA, respectively, found in ICs in SLE [90]. Upon internalization of these ICs, which consist of self‐nucleic acids and autoantibodies, pDCs trigger TLR7/9 signaling pathways, leading to the production of large amounts of IFN‐α [91]. This IFN‐α lowers the activation threshold of B cells, increasing autoantibody production in a positive feedback loop [92]. Notably, pDC activation is further facilitated by the NCF1 variant p.R90H, which enhances the response of pDCs to ICs, exacerbating SLE [93].

The pathogenic role of pDCs has been underscored by studies showing that their depletion in lupus‐prone mice significantly reduces disease severity, autoantibody titers, and kidney pathology [94]. Moreover, pDCs migrate to inflamed tissues such as the kidneys and skin during active SLE phases, where they contribute to local inflammation and tissue damage through sustained IFN‐α production [95]. Chronic pDC activation via TLR7/9 also renders them resistant to regulatory mechanisms such as NF‐κB inhibition, leading to persistent inflammation and steroid resistance in SLE patients [96]. Additionally, the coordinated expression of distinct amino acid transporters is essential for pDC activation, indicating complex metabolic regulation of their function [97].

##### 4.1.2.2 Conventional Dendritic Cells

cDCs, particularly the cDC1 and cDC2 subsets, are involved primarily in presenting antigens and activating T cells. In SLE, cDCs capture, process and present self‐antigens such as nucleoprotein complexes (NPCs) from apoptotic cells to T cells through major histocompatibility complex (MHC) class II molecules, leading to autoreactive T‐cell activation [98, 99]. This antigen presentation is critical for the formation of ectopic lymphoid structures in the inflamed tissues of SLE patients, where they facilitate autoantibody production and sustain chronic inflammation [100].

cDCs in SLE exhibit increased expression of costimulatory molecules and produce proinflammatory cytokines such as IL‐6, IL‐12, and TNF‐α, driving naive CD4+ T cells to differentiate into T helper (Th) cells, especially Th1 and Th17 subsets [99]. Th17 cells, in particular, promote the production of inflammatory cytokines such as IL‐17, contributing to tissue damage and autoimmunity [35]. Additionally, cDCs induce follicular helper T (Tfh) cells, which are crucial for B‐cell assistance and high‐affinity autoantibody production [101]. Recent studies have revealed that cDCs contribute to LN by presenting autoantigens in the kidney: single‐cell profiling has identified CD163+ cDCs in patients with LN, emphasizing their role in renal inflammation [102]. In addition, abnormal DCs in the skin immune microenvironment have attracted attention in the academic community. Similar to the abnormal basal keratinocytes of patients, DCs in the skin lesions of model mice are overactivated by type I IFNs derived from keratinocytes, and this overactivation is sufficient to drive the onset of SLE [103]. Another study revealed that DCs migrating from skin lesions to draining lymph nodes (dLNs) are influenced by locally accumulated farnesyl pyrophosphate, which facilitates the survival and germinal center (GC) reactions of these migrating DCs in dLNs by coordinating protein farnesylation and mitochondrial remodeling [103].

Moreover, cDCs from SLE patients are hyperactivated owing to persistent exposure to ICs and other inflammatory stimuli, resulting in an increased ability to stimulate T cells and produce inflammatory cytokines, exacerbating autoimmunity [104]. This hyperactivation is further influenced by altered X chromosome inactivation, which predisposes individuals to autoimmunity, affecting cDC function [105]. Mitophagy inducers that reduce myeloid cell activation and autoantigen presentation have shown potential in alleviating LN, underscoring the importance of controlling cDC activation in SLE treatment [106].

##### 4.1.2.3 Interactions Between pDCs and cDCs

The interaction between pDCs and cDCs is critical in SLE pathogenesis. IFN‐α produced by pDCs modulates cDC function, enhancing their ability to present antigens and activate T cells, creating a potent environment for autoimmune response amplification [104]. Chronic activation of both pDCs and cDCs in SLE contributes to tolerance breakdown and sustained autoimmunity [35, 104]. Single‐cell sequencing studies have revealed cellular heterogeneity in lupus skin lesions, including diverse DC populations, thus highlighting the complexity of their roles in SLE, even before clinical signs [104, 107].

#### 4.1.3 Macrophages

Macrophages are essential for tissue homeostasis because they clear apoptotic cells and regulate inflammatory responses. In SLE, however, defects in macrophage function disrupt this balance, leading to the accumulation of late apoptotic cells. These cells release NPCs that activate TLRs and other pattern recognition receptors, initiating a cascade that drives the production of proinflammatory cytokines such as TNF‐α, IL‐1β, and IFN‐γ, which, in turn, fuel chronic inflammation and autoimmunity [35, 91, 104].

The inflammatory milieu of SLE further skews macrophage polarization toward a proinflammatory M1 phenotype, characterized by elevated cytokine secretion and the promotion of Th1 responses [104]. This skewing is exacerbated by high levels of TNF‐α, IFN‐γ, and granulocyte‒macrophage colony‐stimulating factor, which create a positive feedback loop that perpetuates inflammation [14, 108]. A key player in this process is the TREML4 receptor on myeloid cells, which facilitates TLR7‐mediated IFN production. Studies in TREML4‐deficient lupus‐prone mice have shown that the absence of this receptor results in reduced IFN production and milder disease symptoms [109]. Additionally, UNC93B1 variants have been linked to TLR7‐dependent autoimmunity, and the instability of the UNC93B1 protein decreases contact with TLR7 but overactivates specific TLR7 with a continuous type I IFN response in macrophages [110].

Epigenetic and transcriptomic reprogramming of monocyte subpopulations during active SLE exacerbates macrophage dysfunction, accelerating disease progression [111]. Circulating monocytes in SLE patients, under conditioning by erythrocytes that retain mitochondria, recognize mitochondrial DNA derived from these erythrocytes. This recognition activates multiple inflammatory pathways, leading to the simultaneous production of IFN and mature interleukin‐1ß [112]. scRNA‐seq studies revealed that in LN, infiltrating monocytes transition into phagocytic macrophages, contributing to both tissue damage and chronic inflammation in the kidneys [113, 114]. LPS from the gut has been identified as a trigger for macrophage pyroptosis through the caspase 11‐gasdermin D pathway. These results link organ systemic crosstalk to further uncover the complex macrophage‐mediated inflammation in SLE [115].

The macrophage transcription factor TonEBP has been identified as a key promoter of kidney injury in SLE, acting through damage‐induced signaling pathways [116]. Erythroid mitochondrial retention has also been shown to trigger IFN‐I responses in macrophages [117]. Collectively, these findings underscore the multifaceted role of macrophages in SLE, in which their dysregulation drives both systemic inflammation and organ‐specific damage.

#### 4.1.4 NK Cells

NK cells regulate immune responses via cytokine production and cytotoxic activity. In SLE, particularly in patients with renal involvement, the number and function of NK cells are often reduced because of elevated levels of IFN‐α, which induces NK cell apoptosis [118]. Despite their reduced numbers, the remaining NK cells produce increased levels of proinflammatory cytokines such as IFN‐γ, exacerbating inflammation and kidney damage [104, 119]. Additionally, cytokine dysregulation, particularly involving IL‐2 and IL‐15, further impairs NK cell development and function, promoting the generation of immature NK cells and sustaining inflammation in affected tissues, such as the kidneys [104, 120].

#### 4.1.5 Basophils

Basophils are the least abundant type of leukocyte. Studies in CT‐M8 mice, a novel mouse model, have demonstrated that basophils play a nonredundant role in the development of lupus‐like disease [121]. In patients with LN, the proportion of basophils is significantly lower than in patients without LN, while the identification of CD62L expression on blood basophils may be a potential pretreatment predictor of remission in severe LN patients [122, 123]. In SLE patients, basophils exhibit an activated phenotype characterized by the overexpression of surface markers such as CD203c and HLA‐DR, as well as increased expression of adhesion molecules such as l‐selectin, which facilitates their migration to secondary lymphoid organs (SLOs) [124]. In these organs, basophils interact with B and T cells, promoting the differentiation of Th2 cells and the production of autoantibodies. For example, IgE autoantibodies in the serum of SLE patients have been shown to activate basophils, leading to further increases in B‐cell activity and autoantibody production, particularly in patients with LN [104]. A study using a mouse model of SLE demonstrated that basophils aggregated in SLOs cooperate with B cells to produce autoantibodies by the production of MHC class II molecules and the membrane‐bound form of B‐cell‐activating factor (BAFF) [104]. Basophils have also been shown to produce IL‐4 and IL‐6 in response to IgE‐dependent and IgE‐independent stimulation, contributing to the inflammatory milieu in SLE and supporting the expansion of autoreactive B and T cells [123, 124]. Moreover, basophils are involved in promoting the accumulation and maintenance of pathogenic Tfh cells, particularly the Tfh2 subset, within SLOs. This process is mediated through the upregulation of PD‐L1 and the production of IL‐4 by basophils, which are crucial for Tfh cell function and the subsequent exacerbation of autoimmunity and LN [125].

#### 4.1.6 MAIT Cells and ILCs

ILCs, which resemble T cells but lack T‐cell receptors, are vital for regulating both adaptive and innate immune responses through cytokine production, contributing to functions such as mucosal immunity, tissue repair, and the maintenance of overall homeostasis. These cells are categorized into five subgroups: ILC1s, ILC2s, ILC3s, NK cells, and lymphoid tissue‐inducer cells [104, 126]. In the context of SLE, a notable increase in ILC1 and ILC3 populations in the peripheral blood has been observed, whereas ILC2 levels significantly decrease, especially during more severe stages of the disease [127, 128]. However, a different investigation revealed that ILC3 levels in SLE patients with moderate disease activity are similar to those in healthy individuals, but ILC3 levels in patients with severe disease activity are reduced [127]. Recent studies using high‐resolution single‐cell analysis of renal immune and parenchymal cells have shown that tissue‐resident NKp46+ ILC1s promote parenchymal cell damage by facilitating the entry of monocyte‐derived macrophages into the renal epithelial cell microenvironment [129]. Additionally, MAIT cells, a distinct subset of T cells with functions akin to those of ILCs, exhibit altered phenotypes and reduced numbers in SLE, particularly in patients with renal involvement. This alteration in MAIT cells is correlated with the severity of the disease and its outcomes [130, 131]. The functions of these cells in SLE are still unclear and require further study.

### 4.2 Adaptive Immunity

The overactivation of T cells is a central mechanism in the pathogenesis of SLE. These aberrant T cells are coupled with APCs presenting self‐antigens, thereby activating autoreactive B cells and promoting the production of autoantibodies. Once the autoreactive adaptive immune response is initiated, persistent exposure to self‐antigens and defects in immune tolerance mechanisms sustain the autoimmune reaction over time, ultimately leading to organ damage. Although the upstream mechanisms of SLE pathogenesis remain largely unknown, the pronounced phenotypic alterations and critical pathological roles of T‐ and B‐cell abnormalities, which are central to the downstream phases of the autoimmune response, make them pivotal targets in translational research. In this review, we summarize the roles of adaptive immune cells in the pathogenesis of SLE.

#### 4.2.1 T Cells

Various subsets of T cells contribute to the complex immunological landscape in SLE. Alterations in T‐cell signaling, cytokine production, proliferation, and regulatory functions are well documented in SLE patients. The following sections detail the specific roles of different T‐cell subsets in SLE.

##### 4.2.1.1 Th1 and Th2 Cells

Th1 and Th2 cells are pivotal CD4+ T‐cell subsets that orchestrate distinct immune responses. In SLE, the delicate balance between Th1 and Th2 cells is often disrupted. Th1 cells, which produce IFN‐γ, are instrumental in driving proinflammatory responses. SLE‐associated genetic variants, such as those affecting the STAT4 gene, have been linked to increased IFN‐γ production in T cells, which exacerbates autoimmune responses [132]. In experimental models, increased STAT4 activity was shown to upregulate IFN‐γ, leading to increased macrophage activation and subsequent tissue damage [133]. Moreover, Th2 cells secrete cytokines such as IL‐4, IL‐5, and IL‐13, which are traditionally associated with humoral immunity and allergic responses. Although their role in SLE is less defined, Th2 cytokines can enhance B‐cell activation and promote the production of pathogenic autoantibodies [14, 104]. Studies have demonstrated that in lupus‐prone mice, IL‐4 contributes to the skewing of immune responses toward autoantibody production, further aggravating the disease [134].

##### 4.2.1.2 Th17 Cells

Th17 cells, a distinct subset of CD4+ T cells, are potent producers of the proinflammatory cytokine IL‐17. Th17 cells have been shown to exacerbate SLE by altering both humoral and cellular immune responses. IL‐17 promotes the activation of B cells and DCs, leading to increased antibody production and an intensified inflammatory response in target tissues such as the kidneys. In mouse models of LN, increased levels of IL‐17 are directly associated with severe renal inflammation and damage, highlighting the pathogenic role of Th17 cells in SLE [14, 104]. Furthermore, clinical studies in SLE patients have revealed that Th17 cell frequencies are significantly elevated, particularly in those with active renal disease, which is correlated with increased disease activity [135]. The involvement of Th17 cells in SLE is further underscored by findings that SIRT2, a deacetylase, modulates IL‐17A transcription. The inhibition of SIRT2 in lupus‐prone mice reduces IL‐17A levels and ameliorates disease symptoms, demonstrating a direct link between Th17 cell activity and SLE pathogenesis [136].

##### 4.2.1.3 Regulatory T Cells

Tregs are essential for maintaining immune tolerance and preventing autoimmunity by suppressing overactive immune responses. In SLE, both the number and function of Tregs are often compromised, leading to unchecked immune activation. Tregs are characterized by the expression of the transcription factor FOXP3 and the high‐affinity IL‐2 receptor alpha chain (CD25) [135]. Experimental evidence suggests that in SLE, there is a marked decrease in Treg suppressive function, partially caused by downregulation of FOXP3, which results in reduced inhibition of autoreactive T cells. Studies using lupus‐prone mouse models have shown that Treg deficiency or dysfunction leads to the exacerbation of autoimmune symptoms, including increased autoantibody production and more severe organ damage [135, 137]. Furthermore, the metabolic regulation of Tregs is crucial for their stability and function. Phosphofructokinase P is a key regulator of Treg metabolism, and its dysregulation can impair Treg function, destabilizing immune tolerance in autoimmune conditions such as SLE [138]. Restoring Treg function through low‐dose IL‐2 administration has exhibited potential ability to improve Treg proliferation and immune regulation in SLE patients [14]. Additionally, platelets can impair Treg cell function by forming aggregates through P‐selectin on platelets and PSGL‐1 on Treg cells, a process that occurs more frequently in individuals with active SLE than in healthy donors. Blocking the P‐selectin/PSGL‐1 interaction reduces disease severity in Dnase1l3‐KO lupus‐prone mice [139].

##### 4.2.1.4 T Follicular Helper Cells

Tfh cells are a specialized subset of CD4+ T cells that play crucial roles in the formation of GCs and the differentiation of B cells into plasma cells and memory B cells. Tfh cells produce IL‐21, which is essential for B‐cell differentiation and the production of high‐affinity antibodies [35, 135]. In SLE, an expansion of Tfh cells is commonly observed, which correlates with elevated levels of pathogenic autoantibodies [140]. Dysregulation of Tfh cells is driven by various factors, including the overexpression of costimulatory molecules such as inducible costimulator (ICOS), which enhances Tfh cell survival and function. For example, studies in mice have shown that the ubiquitin ligase Peli1 regulates Tfh responses by inhibiting ICOS, thus modulating autoimmune responses [141]. Conversely, deficiencies in CBL and CBLB ubiquitin ligases lead to hyperactive Tfh responses and lupus development by reducing the degradation of BCL6, a transcription factor critical for Tfh cell function [142]. Additionally, the NLRP3 inflammasome has been shown to be essential for Tfh cell function, particularly in the context of high‐affinity antibody generation and GC formation, contributing to the autoimmune processes observed in SLE [143]. The accumulation of Tfh cells in SLE is further promoted by basophils expressing PD‐L1 and IL‐4, which create a supportive environment for Tfh cell expansion and autoantibody production [125].

##### 4.2.1.5 T Peripheral Helper Cells

T peripheral helper (Tph) cells are a recently identified subset of CD4+ T cells that, like Tfh cells, are important in supporting B‐cell differentiation and antibody generation. However, unlike Tfh cells, Tph cells are found in the circulation and do not express CXCR5, which is a hallmark of Tfh cells [144]. Tph cells are notably enriched in the blood and potentially in the skin and kidneys of SLE patients with or without nephritis [145, 146]. These cells produce IL‐21, IFN‐γ and IL‐10, all of which support B‐cell function and enhance local inflammation, leading to tissue damage [144, 147]. A recent study revealed that the expansion of CXCL13+ Tfh and Tph cells, alongside a reduction in CD96hi IL‐22+ T cells, is a key imbalance in T‐cell phenotypes in SLE patients. The aryl hydrocarbon receptor (AHR) acts as a potent regulator, inhibiting CXCL13 production while promoting an IL‐22+ T‐cell phenotype [148, 149]. Conversely, IFN‐I opposes AHR to increase CXCL13 production, positioning AHR, JUN, and IFN as central regulators of these divergent T‐cell states [148].

##### 4.2.1.6 CD8+ T Cells

CD8+ T cells, known for their cytotoxic functions, are also critically involved in SLE pathogenesis. Early in the disease, the number of CD8+ T cells is often increased, particularly in the kidneys of patients with LN [70, 135]. However, these cells frequently exhibit impaired cytotoxic function, which contributes to the increased susceptibility to infections observed in SLE patients [35, 150]. Studies have shown that CD8+ T cells in SLE patients produce lower levels of granzymes and perforin, key molecules required for effective cytotoxic responses, which diminishes their ability to control viral infections and other immune challenges [151]. Furthermore, these cells often express markers of exhaustion, such as CD38, which further reduces their cytotoxic capacity [151, 152]. CD38 has been found to suppress mitophagy, leading to reduced mitochondrial fitness and weakened cytotoxic responses in lupus patients [153]. Additionally, type I IFNs, which are abundantly produced in SLE, disrupt the metabolic fitness of CD8+ T cells, further impairing their function and increasing cell death [154]. The identification of specific CD8+ T‐cell subsets, such as the CD8+ CD27+ CXCR3− population, which is dysregulated in SLE, has provided new insights into the mechanisms driving disease progression and highlights the potential for these cells to serve as biomarkers for disease severity [155]. Therapeutic strategies aimed at restoring CD8+ T‐cell function, such as inhibiting CD38‐mediated NAD degradation, may offer significant benefits for patients with SLE [104].

#### 4.2.2 B Cells

B cells function as precursors to autoantibody‐producing plasma cells and as APCs that activate T cells. Dysregulation of the B‐cell tolerance checkpoint leads to the survival and activation of autoreactive B cells, which are essential for the production of autoantibodies [156, 157]. In addition, in patients with NPSLE, blood‒brain barrier disruption facilitates the penetration of B cells into the hippocampus, augmenting inflammation and contributing to early diffuse neuropsychiatric symptoms [158].

Age‐associated B cells (ABCs), marked by IgD‐CD27‐ and CD21low expression, have been identified as critical contributors to SLE pathogenesis [157]. Unlike typical B cells, ABCs proliferate in response to TLR7 and TLR9 stimulation rather than B‐cell receptor (BCR) cross‐linking [159]. In SLE, these cells are generated in response to RNA‐containing ICs and the activation of pDCs [14]. These cells are regulated by transcription factors such as IRF5, which drive their proliferation and autoantibody generation through IL‐21 expression and chromatin remodeling [160]. The transcription factor ZEB2 promotes the formation of ABCs, further contributing to disease [161].

The molecular signature of SLE B cells is often established early during the naive phase, marked by the enrichment of chromatin motifs for transcription factors such as AP‐1 and EGR, which are likely influenced by IFN‐γ signaling [157, 162]. These B cells, particularly those lacking IgD and CD27, exhibit hyperresponsiveness to TLR7 agonists and IL‐21, leading to increased autoantibody production [163]. Recent findings have also demonstrated that gain‐of‐function mutations in TLR7 can drive disease in humans by promoting excessive B‐cell activation and autoantibody production [164].

SLE patients often exhibit elevated levels of IgE antibodies specific for dsDNA. These IgE antibodies can activate pDCs, leading to significant IFN‐α secretion and further exacerbating the autoimmune response [165]. Additionally, B1 cells, another subset of B cells, have been implicated in LN through the production of antiphosphatidylserine antibodies, which activate the TLR‐mediated Syk signaling pathway, thereby driving kidney inflammation [166].

Tfh cells are important for promoting B‐cell differentiation and high‐affinity IgG autoantibody production, particularly in SLE. Dysregulation of Tfh cell function, driven by costimulatory molecules such as ICOS and OX40L, is a key factor in the pathogenic autoantibody response observed in SLE [14, 104, 144]. The expansion of Tfh cells in SLE patients is often resistant to inhibitory signals, such as those mediated by the P2X7 receptor, suggesting defects in the signaling pathways that typically restrain their activity [167]. This dysregulation is further compounded by the presence of basophils expressing PD‐L1 and IL‐4, which promote Tfh cell accumulation and sustained autoantibody production [125].

Plasmablasts and long‐lived plasma cells are expanded in SLE, highlighting the persistent nature of autoantibody production. Plasmablasts, which express CD19, can be targeted by therapies such as anti‐CD19 chimeric antigen receptor (CAR)‐T cells. However, long‐lived plasma cells, which are responsible for producing autoantibodies against nuclear antigens such as anti‐Sm and anti‐RNP, lack CD19 expression and thus evade such treatments [168].

In addition, innate‐like B cells, which produce natural IgM antibodies crucial for clearing apoptotic cells, are deficient in SLE patients. This deficiency leads to the persistence of autoreactive B cells, exacerbating the autoimmune response [14]. IFN‐I may impair B‐cell function by increasing the expression of IFN‐I‐stimulated genes in bone marrow B cells and their recent emigrants [14, 169]. IFN‐I may disrupt B‐cell self‐tolerance by increasing the sensitivity of BCR signaling, reducing the activation threshold needed for B cells to respond to antigens and thus promoting autoreactive B‐cell development into the antibody‐forming cell and GC pathways [170].

Recent findings underscore the importance of metabolic processes in modulating B‐cell function. Oxidative phosphorylation is a crucial metabolic process that supports B‐cell activation and function. Inhibitors such as IM156 have shown potential in suppressing B‐cell activation by regulating the mitochondrial membrane potential, thereby mitigating symptoms of SLE [171]. NADPH oxidase plays an intrinsic role in B cells by modulating endosomal TLR signaling pathways. In lupus‐prone mice, a deficiency in NADPH oxidase has been shown to increase TLR7 and TLR9 signaling, resulting in increased autoantibody production, which exacerbates lupus risk [172]. Another study revealed that the methyltransferase METTL1 catalyzes m7G modification in specific subsets of tRNAs, preferentially translating BCR signaling‐related proteins. This modification ensures the activity of the mitochondrial electron transport chain and sufficient bioenergy in B cells, thereby promoting the GC reaction and exacerbating the autoimmune response [173]. Furthermore, thioredoxin has been recognized as a crucial factor in regulatory B cells (Bregs). Dysregulation of thioredoxin in lupus can impair Breg function, leading to immune imbalance and promoting autoimmunity [174].

## 5 New Therapeutic Targets

**FIGURE 3:**
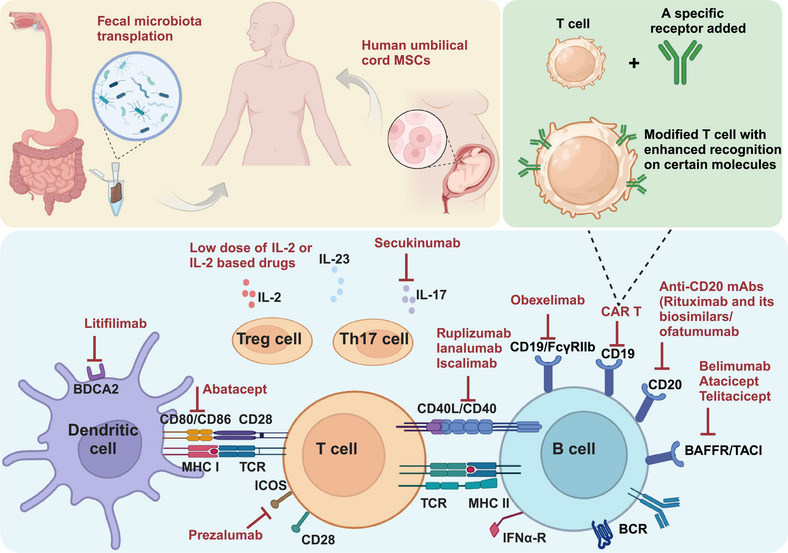
Promising therapeutic targets confirmed with clinical trials for SLE. This figure presents an overview of the treatment strategies for SLE, divided into targeting innate immunity such as dendritic cells, targeting adaptive immunity such as B cells and T cells, targeting cytokines and signaling pathways such as IL‐2 and IL‐17, and novel treatments like fecal microbiota transplantation and human umbilical cord MSCs. Innovative CAR‐T strategy is illustrated. BAFFR, B‐cell‐activating factor receptor; BCR, B cell receptor; BDCA 2, blood dendritic cell antigen 2; CAR‐T, chimeric antigen receptor T; ICOS, inducible costimulator; IL‐23, interleukin 23; MHC, major histocompatibility complex; TACI, transmembrane activator and CAML interactor; TCR, T cell receptor.

As the comprehension of SLE pathogenesis deepens, novel therapeutic targets have emerged as potential strategies for more effective treatment of the disease. Although current strategies are helpful in controlling symptoms, they may be limited by issues such as side effects and inconsistent efficacy. Therefore, there is an unmet need for novel therapies with more precise modulation. This section summarizes these emerging targets in innate immunity, adaptive immunity, cytokine and signaling pathway and other novel treatments (Figure 3). Both completed preclinical and clinical trials (Table 1) as well as those under investigation (Table 2) will be reviewed, providing a comprehensive and updated perspective on the advances in SLE treatment.

**TABLE 1:** Completed preclinical or clinical study on therapeutic targets for SLE in recent 5 years.

Target/agent	Model/disease	Primary outcome (clinical)/main outcome (preclinical)	Whether primary outcome was achieved (for clinical trials)	Category
**Targeting innate immunity**				
Targeting pDCs via BDCA2 (litifilimab)				
	SLE	Swollen/tender joint count change at week 24	Yes	Phase II study [15]
**Targeting adaptive immunity**				
A CD19 mono‐antibody–drug conjugate				
	MRL/lpr mice/human PBMC	Enhanced effectiveness in depleting B cells, alleviated lupus symptoms	Not applicable	Preclinical study [175]
Anti‐CD19/FcγRIIb (obexelimab)				
	SLE‐prone B6.hRIIb and NZM.hRIIb mice	Inhibition of B cell activation in spleen and lymph nodes for over two weeks	Not applicable	Preclinical study [176]
	Moderate‐to‐severe non‐major organ SLE	Week 32 sustained response rate	No	Phase II, double‐blind, multicenter study [177]
Anti‐CD20 (obinutuzumab)				
	MRL/lpr mice	Superior B‐cell depletion efficacy than RTX; effective in early‐stage disease (reduced glomerulonephritis, anti‐RNA autoantibody titers and activated CD4 T cells); effective in advanced disease and prolonged survival	Not applicable	Preclinical study [178]
Anti‐CD20 (abinutuzumab)				
	SLE patients	Proportion of patients achieved CRR at week 52	Yes	Phase II study [179]
Anti‐BAFF/APRIL(povetacicept)				
	Lupus mice and non‐human primates	Improved survival and reduced disease markers (proteinuria, anti‐dsDNA antibodies, and key immune cell populations) in NZB × NZW and BM 12 mice; decreased serum immunoglobulins and well tolerated in primates	Not applicable	Preclinical study [180]
	Healthy adults	Type, incidence, and severity of TEAEs over a 30‐day period	Yes	Phase I study [181]
**Signaling pathway modulation**				
Fenebrutini (BTK inhibitor)				
	Severely active SLE	SRI‐4 response rates for fenebrutinib (150 mg once daily: 51%, 200 mg twice daily: 52%) were not significantly better than placebo (44%)	No	Phase II, double‐blind, multicenter study [182]
**Novel approaches**				
Anti‐CD19 CAR‐T				
	Refractory SLE	No disease activity for long observation (up to 29 months) without obvious AEs	Not applicable	Case report [183, 184, 185]
	CNS‐SLE	Improved physical function in SLE‐related CNS inflammation	Not applicable	Case report [186]
LNP023 (complement factor B inhibitor)				
	Lupus mice	Renal function amelioration, serum anti‐dsDNA and ANA antibody titers reduction, renal immunoglobulins and complements deposition reduction	Not applicable	Preclinical study [187]

**TABLE 2:** Ongoing clinical trials on therapeutic targets for SLE.

Mechanism	Main outcome	Category
**Targeting innate immunity**		
Litifilimab (targeting pDCs via BDCA2)		
	SRI‐4 response rate at week 52	Phase III, double‐blind, multicenter study [188]
	SRI‐4 response rate at week 52	Phase III, double‐blind, multicenter study [189]
	Incidence of TEAEs and SAEs up to week 180	Phase III, double‐blind, multicenter study [190]
E6742 (TLR7/TLR8 inhibitor)		
	Incidence of TEAEs and SAEs up to approximately 1 year 5 months	Phase I/II, double‐blind, multicenter study [191]
**Targeting adaptive immunity**		
Anti‐CD19/FcγRIIb (obexelimab)		
	The proportion of SLE patients achieving BILAG‐Based Composite Lupus Assessment (BICLA) Response at week 24	Phase II, double‐blind, multicenter study [192]
Anti‐CD20 (obinutuzumab)		
	Percentage of participants with CRR for Class III or IV lupus nephritis at week 76	Phase III, double‐blind, multicenter study [193]
Anti‐BAFF/APRIL (telitacicept)		
	Week 24 LLDAS achievement criteria (proportion of patients): SLEDAI‐2k≤4; no major organ involvement; no new disease manifestations; PGA score≤1 (0–3 scale); prednisone ≤7.5 mg/day; permitted maintenance IS/antimalarials	Phase IV, double‐blind, multicenter study [194]
	SRI‐4 response rate at week 24	Phase IV, open‐label, single‐center study [195]
	SRI‐4 response rate at week 24	Phase IV study [196]
Sequential B cell‐targeted therapies (belimumab plus rituximab)		
	To evaluate the efficacy of B‐cell‐targeted combination therapy in reducing treatment failure rates	Phase III, open‐label, multicenter study [197]
**Cytokine and signaling pathway modulation**		
IL‐2 combined other agents		
IL‐2 plus human umbilical cord MSCs		
	Complete response rate and partial response rate at week 24	Phase III, open‐label, single‐center study [198]
IL‐2 plus telitacicept		
	SRI‐4 response rate at week 24	Phase III, open‐label, single‐center study [199]
IL‐2 plus belimumab		
	B cell subsets immune response at week 24	Phase III, open‐label, single‐center study [200]
IL‐2 analogues		
CUG252	TEAEs incidence through 10 weeks	Phase I, double‐blind, single‐center study [201]
CD40L inhibitor		
Dapirolizumab pegol	TEAEs, SAEs and discontinuation‐related TEAEs incidence with dapirolizumab pegol	Phase III, double‐blind, study [202]
Baricitinib (targeting JAK–STAT pathways)		
	24 h protein in urine	Phase III, double‐blind, single‐center study [203]
Deucravacitinib (tyrosine kinase 2)		
	SRI‐4 response rate at week 52	Phase III, double‐blind, multicenter study [204]
Anifrolumab (anti‐IFNα receptor mAb)		
	To define the PK characterization and the dose of anifrolumab in pediatric moderate to severe active SLE up to day 29; BICLA response count at week 52	Phase III, double‐blind, multicenter study [205, 206]
	Anifrolumab versus placebo CRR rate difference in active proliferative LN	Phase III, double‐blind, multinational study [205, 206]
**Novel approaches**		
Fecal microbiota transplantation		
	SRI‐4 response rate over a 6‐month period	Phase II, double‐blind, multicenter study [207]
CAR‐T therapy		
Anti‐CD19 CAR‐T		
	To access the incidence of DLTs across 4 dose levels over a 3‐month period for moderate or severe active SLE and to determine a phase II recommended dose	Phase I, open‐label, single‐arm, multicenter study [208]
	Recommended dose out of 3 dose levels 28 days later after infusion (Phase I) incidence of AEs, evaluation and classification of CRS and ICANS for 24‐month period (Phase I); 6‐month postinfusion remission rate (Phase IIa)	Phase l/ll, open‐label, single‐arm, multicenter study [209]
Anti‐CD19 CAR–NK cell therapy		
	Incidence of AEs including DLT from first dose over a 2‐year period	Phase I, open‐label, single‐center study [210]
	DLT incidence within 28 days after infusion and incidence of AEs over a 12‐month period	Phase I, open‐label, single‐center, single‐arm study [211]
CD19/CD20 CAR‐T therapy		
	Incidence of DLTs, SAEs, and TEAEs for active and refractory SLE within 28 days after infusion	Early Phase I, open‐label, single‐center study [212]
	The rate of DLT within 28 days after infusion, the rate of AEs and SAEs up to 2 years	Phase I, open‐label, single‐arm, single‐center study [213]
Anti‐BCMA/CD19 CAR‐T therapy		
	Incidence and severity of DLTs for relapsed/​refractory SLE over a 28‐day period	Phase I/II, open‐label, single‐arm, single‐center study [214]
	Incidence and severity of DLTs for ​refractory SLE over a 28‐day period	Phase I/II, open‐label, single‐arm, single‐center study [215]
Anti‐CD19–CD3E–CAR‐T therapy		
	Incidence and severity of DLTs and proportion of relapsed/refractory SLE patients in whom successful manufacturing of the desired dose of anti‐CD19–CD3E–CAR‐T cells is achieved; clinical response for relapsed/refractory SLE 12 weeks post infusion	Phase I, open‐label, single‐arm, single‐center study [14]
CD19–CAR–DNT cells therapy		
	To evaluate DLTs, maximum tolerated dose and incidence of AE/SAE/AESI/laboratory tests/electrocardiograms/vital signs up to 28 days	Phase I, open‐label, single‐arm, multicenter study [216]
LNP023 (an alternative complement inhibitor)		
	Proportion of participants with CRR at week 24 in the absence of renal flares	Phase II, double‐blind, multicenter study [217]

Abbreviations: AEs, adverse events; AESI, adverse events with special interest; BICLA, British Isles Lupus Assessment Group‐based Composite Lupus Assessment; CRR, complete renal response; CRS, cytokine release syndrome; DLTs, dose‐limiting toxicities; ICANS, immune effector cell‐associated neurotoxicity syndrome; LN, lupus nephritis; SAEs, serious adverse events; SRI‐4, SLE Responder Index 4; TEAEs, treatment‐emergent adverse events.

### 5.1 Targeting Innate Immunity

Innate immunity is central to SLE immunopathogenesis, with pDCs and TLRs serving as important drivers of inflammatory responses. This section explores therapeutic strategies targeting these innate immune components, specifically ​pDCs and the ​TLR7/8 signaling pathway, to modulate excessive immune activation.

#### 5.1.1 Targeting pDCs

Litifilimab (BIIB059), a humanized IgG1 monoclonal antibody directly targeting blood dendritic cell antigen 2 (BDCA2), has emerged as a promising therapeutic agent for SLE. This pDC‐specific receptor inhibitor demonstrated clinically meaningful efficacy in a phase II trial, where SLE patients receiving litifilimab showed sustained reduced joint inflammation for 24 weeks compared with placebo [15]. Litifilimab also showed some efficacy in a phase II trial in SLE using specific skin indicators, demonstrating its potential for SLE treatment. Three phase III trials (NCT04961567 [188], NCT04895241 [189], and NCT05352919 [190]) of litiflimab for SLE are being conducted.

#### 5.1.2 Targeting TLR7/8

TLR7, which is abundant on the surface of pDCs, plays a crucial role in initiating the production of IFN‐I in SLE. TLR8, which is more highly expressed in neutrophils, monocytes, and myeloid DCs, produces inflammatory cytokines by activating the NF‐κB pathway. The effect of E6742, a dual inhibitor of TLR7 and TLR8, is stronger than that of either one alone in lupus mice. In NZB/W mice, mortality and proteinuria were decreased by E6742, and weight loss and nephropathy progression were inhibited 5 months later. In pristane‐induced lupus mice, E6742 decreased arthritis signs and suppressed the increase in antiribosomal P protein in the plasma at 3 months postadministration [218]. A phase I trial [219] in healthy adults confirmed that E6742 was safe and tolerated in a single ascending dose study and in a multiple ascending dose. In this trial, E6742 was applied in SLE patients in a completed phase I/II trial (NCT05278663 [191]), but the results have not yet been revealed.

### 5.2 Targeting Adaptive Immunity

Recently, numerous studies on the mechanisms underlying SLE and corresponding treatment regimens have been reported. However, the majority of these studies are still in the phase I or II clinical trial stage. Strategies specifically targeting adaptive immunity, such as B‐cell activation and T‐cell regulation, are likely to have accumulated more extensive clinical evidence.

#### 5.2.1 Targeting B Cells

**FIGURE 4:**
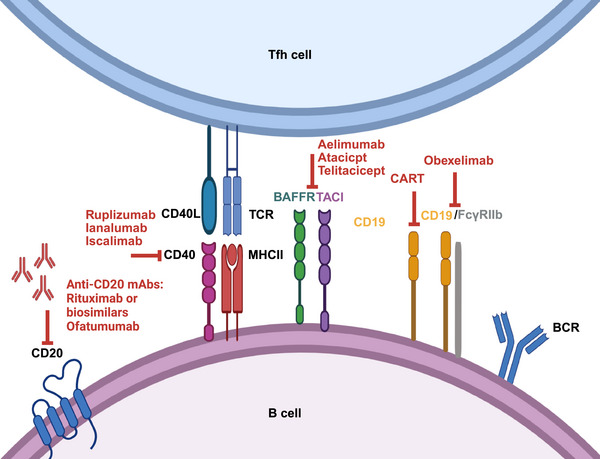
Promising therapeutic targets B cells in SLE. B cell‐targeted strategies for SLE include anci‐CD19 (targets early and memory B cells), anti‐CD20 therapy (depletes mature B cells), anti‐CD40/CD40L therapy (interferes with B cell activation and autoantibody production), and sequential therapies (combine two or more agents to boost efficacy). CD40L, CD40 ligand; mAbs, monoclonal antibodies.

In the context of B‐cell‐targeted strategies for SLE, treatments can generally be classified into the following approaches: anti‐CD19 therapy, which targets early and memory B cells; anti‐CD20 therapy, which depletes mature B cells; anti‐CD40/CD40L therapy, which interferes with B‐cell activation and autoantibody production; and sequential therapies, which combine different immunotherapeutic agents to increase overall treatment efficacy (Figure 4).

##### 5.2.1.1 | Anti‐CD19

CD19 is a membrane‐bound protein found on the surface of all B lymphocytes. It serves as a coreceptor in BCR signaling to facilitate the activation, signal transduction, and development of B cells. Anti‐CD19 therapy is a promising strategy for depleting B cells. However, the use of an anti‐CD19 antibody alone is still in the preliminary stage of exploration and is insufficient to eliminate B cells. A newly constructed antibody‒drug conjugate targeting CD19 incorporating triptolide, a bioactive component from traditional SLE treatment Tripterygium wilfordii Hook F, exhibited excellent therapeutic CD19+ B‐cell depletion efficacy in MRL/lpr mice in vivo and in human PBMCs in vitro [175]. Other studies targeting CD19 have involved more innovative strategies, such as CD19/FcγRIIb monoclonal antibodies and allogeneic CAR‐immune cell therapies.

Obexelimab (XmAb5871), which targets CD19/FcγRIIb, is a bispecific monoclonal antibody consisting of an antibody construct with a CD19‐specific variable domain and an FcγRIIb‐binding Fc domain on B cells [220]. Mechanically, obexelimab can limit calcium transfer, cell proliferation, and B‐cell costimulatory molecules expression, imitating the capacity of antigen‒antibody complexes to inhibit B‐cell activity, thus suppressing humoral immunity and reducing antibodies [221]. This agent coengages BCR and FcγRIIb, making splenic and lymph node B cells hyporesponsive to BCR activation for more than two weeks in SLE‐prone B6.hRIIb and NZM.hRIIb mice, which were genetically modified to express the human FcγRIIb extracellular domain [176]. A phase II study revealed that B cells decreased by approximately 50% in the obexelimab therapy group, with a notable increase in the time to loss of improvement of 32 weeks in moderate‐to‐severe non‐organ‐threatening SLE patients [177]. Temporary adverse reactions and infusion‐associated gastrointestinal symptoms occurred at rates comparable to those associated with the placebo [177]. An ongoing phase II, multicenter, randomized, double‐blind, placebo‐controlled trial [192] consists of a 24‐week treatment period followed by a 12‐week follow‐up period and aims to evaluate the proportion of SLE patients attaining the BILAG‐Based Composite Lupus Assessment (BICLA) defined response. However, whether such an anti‐CD19/FcγRIIb strategy can achieve a favorable benefit‐risk profile still awaits more clinical trials.

##### 5.2.1.2 | Anti‐CD20

Rituximab (RTX) is a CD20‐directed monoclonal antibody of mouse‒human chimeric origin. It eliminates B cells by targeting CD20 while preserving precursor B cells, pro‐B cells, or plasma cells [222]. RTX was initially approved for lymphoma [223]. While RTX missed the main outcomes of overall cutaneous response at months 6 and 12 and renal response at 52 weeks in the EXPLORER and LUNAR trials, respectively [224, 225], several real‐world studies have supported its efficacy in refractory SLE [226, 227]. RTX has been administered beyond the approved indications for more than two decades and is recommended for refractory SLE and LN by the EULAR [228]. In a meta‐analysis enrolling 1112 patients, the SLEDAI and BILAG scores decreased substantially; 72% of SLE patients achieved an overall global response, and 46% achieved a complete response [226]. SLE patients exhibit a reduced prednisone dosage and alleviated proteinuria after RTX treatment [226]. Notably, B‐cell depletion by RTX is thought to inherently increase infection risk, whereas the rates of AEs or severe adverse events (SAEs) showed no marked variations between the B‐cell‐depleted group and the placebo group [229]. More affordable RTX biosimilars, which share highly similar structures and functions, such as RTX‐abbs [230], RTX‐arrx [231] and RTX‐pvvr [232], have been developed as alternatives. In a retrospective study, a decrease in the modified SLEDAI score ≥4 was achieved by RTX‐abbs in refractory SLE patients at 6 months [230]. The efficacy, safety, and accessibility of RTX biosimilars for SLE still require further validation.

Type I anti‐CD20 mAbs like RTX induce complement‐dependent cytotoxicity, but undergo rapid internalization, leading to limited complete renal response (CRR) SLE patients [233]. Conversely, obinutuzumab, a type II anti‐CD20 mAb, features an afucosylated Fc portion that enhances antibody‐dependent cellular cytotoxicity [234], exhibiting greater efficacy in depleting B cells than type I anti‐CD20 mAbs across early and late phases in murine lupus [178]. While obinutuzumab has been a first‐line therapy for follicular lymphoma [235, 236], a preclinical animal trial in lupus mice confirmed that obinutuzumab greatly relieved renal damage, lowered anti‐RNA autoantibodies and activated CD4 T cells compared with RTX [178]. A retrospective study revealed that all nine RTX‐nonresponsive SLE patients who switched to obinutuzumab achieved obvious reductions in median disease activity scores and complement C3 and dsDNA titers at 6 months [237]. Among these nine patients, six achieved complete response [237]. The NOBILITY trial revealed a higher proportion of patients achieving CRR with obinutuzumab than placebo (35% [22 out of 63] versus 23% [14 out of 62]) [179]. These promising results led to further testing of obinuzumab in a global phase III trial for LN [193].

Ofatumumab is another candidate human anti‐CD20 mAb for lupus that has been approved for chronic lymphocytic leukemia and multiple sclerosis [238, 239, 240]. Its unique epitopes facilitate the binding of CD20 at both the small extracellular loop and the N‐terminus of the large extracellular loop, distinguishing it from obinutuzumab [241]. Accordingly, ofatumumab exhibits greater complement‐dependent and antibody‐dependent cell‐mediated cytotoxicity than obinutuzumab in leukemia [238]. Its benefits, such as proteinuria reduction, autoantibody and disease activity control for SLE, have been shown in scattered cases [242, 243, 244], but rigorous clinical trials are lacking.

##### 5.2.1.3 | Anti‐BAFF or anti‐BAFF/APRIL

BAFF, also known as Blys (B lymphocyte stimulator), and APRIL (a proliferation‐inducing ligand) play crucial roles in B cell survival, activation, and differentiation. This section focuses on therapies that target BAFF or both BAFF and APRIL.

Belimumab, a humanized immunoglobulin G1λ monoclonal antibody that inhibits BAFF, has been recommended to control SLE, facilitate glucocorticoid tapering/discontinuation and serve as a first‐line therapy in severe SLE patients without major organ involvement [222, 228]. Materne et al. [245] recently analyzed data from SLE patients without LN in a US database from the last decade and reported a lower risk of infection with belimumab than with methotrexate and lower risks of serious infection and hospitalization than with azathioprine and mycophenolate.

Atacicept, telitacicept and povetacicept are recombinant fusion proteins, functioning as soluble TACI receptors (transmembrane activator and CAML interactor receptors), which inhibit both BAFF and APRIL—cytokines essential for B‐cell activation and survival.

Atacicept has inconsistent results in different clinical trials. A phase II/III APRIL‐SLE study [246] in 2012 was terminated prematurely because of infection‐related deaths. An improvement was made in the ADDRESS II trial in 2021 [247] when vaccinations against pneumococcus and seasonal influenza were injected prior to atacicept use. In the treatment of up to 7 years, atacicept was rather safe and effective, with lower infection rates than APRIL‐SLE without drug‐related death. This study ceased later because of a medicine shortage.

Telitacicept was granted fast track designation by the United States Food and Drug Administration as a possible treatment for lupus in 2020. It was also approved conditionally in China in March 2021 for active SLE patients who do not respond to conventional treatment. A phase 2b trial [248] from China tested three doses (80, 160, and 240 mg) of telitacicept in active SLE patients and patients achieved higher SLE SRI‐4 response rates with telitacicept than placebo at 48 weeks without obvious AEs or SAEs. A further phase 4 RCT (randomized controlled trial) in China [194] will test it in the active early stage of adult SLE patients whose disease duration is no more than 2 years. Several phase IV single group assignment trials will evaluate telitacicept's therapeutic effects in SLE cohorts in China by utilizing proteomics and metabolomics [195] or analyzing lymphocyte subsets (ChiCTR2400086874 [196]). Hopefully, these further investigations of telitacicept may provide more promising evidence for SLE patients.

Povetacicept is another BAFF/APRIL inhibitor whose superiority over other B‐cell modulators was newly confirmed. In NZB × NZW mice and BM12 mice, povetacicept improved survival, reduced disease markers such as proteinuria and anti‐dsDNA antibodies, and decreased key immune cell populations. Additionally, in nonhuman primates, it was well tolerated and significantly reduced the serum levels of IgM, IgA, and IgG after a single dose [180]. A recent phase I study revealed that the number of antibody‐secreting cells and circulating antibodies were reduced after povetacicept treatment in healthy adults (NCT05034484) [181, 249], suggesting the need for further clinical evaluation.

##### 5.2.1.4 | Sequential B‐Cell‐Targeted Therapies

RTX therapy may fail in some lupus patients, in which case the residual B‐cell number could guide subsequent treatment [229]. In some RTX patients, relapses can be observed after B‐cell depletion, featuring increased anti‐dsDNA antibodies, plasmablasts, and BAFF levels. The early rise in plasmablasts after RTX therapy amplifies Tfh cells, and elevated BAFF stimulates plasmablast and Tfh cell development, leading to further intensification of GC reactions and increased autoantibody production. This loop explains the resistance of SLE to B‐cell depletion [250]. Given this context, sequential B‐cell‐targeted therapies, in which B‐cell‐directed drugs are administered one after another in a planned sequence to achieve the desired therapeutic effect, have become promising.

Kraaij et al. in 2018 [251] conducted the first clinical trial to find improved immunological and clinical responses using combined RTX and belimumab in severe and refractory SLE patients. Shipa et al. [252] reported the effectiveness of belimumab treatment post‐RTX in refractory SLE, with considerable diminishment of serum anti‐dsDNA IgG and a reduced exacerbation risk. The ongoing Synbiose 2 study will test Belimumab plus RTX in severe SLE treatment and introduce a dose‐down process for prednisolone and mycophenolate [197].

#### 5.2.2 Targeting T Cells

##### 5.2.2.1 | Targeting Autoreactive T Cells

Abatacept compromises an extracellular domain of human CTLA‐4 and a human IgG Fc region. By binding to CD80/CD86 on APCs such as DCs and B cells, CTLA‐4 could inhibit costimulatory signals between CD80/CD86 and T‐cell CD28 and thus inhibit T and B‐cell function. However, whether abatacept is effective and safe for treating LN has not been validated. A phase II/III multicenter randomized double‐blind placebo‐controlled trial [253] for treating active LN reported that abatacept groups (both 30 and 10 milligrams per kilogram) presented greater CRRs (22 and 24%, respectively) than placebo groups did (6%). However, this trial ceased because the CRR, which was the primary endpoint even if it fulfilled the secondary endpoint, was missing. ALLURE, a phase III multicenter clinical trial, tested the use of abatacept in active LN patients on standard therapy, but the CRR did not display an obvious distinction between abatacept and placebo. As no clinical trials on abatacept for the treatment of SLE have been carried out in the past 5 years, the effectiveness and safety profile of abatacept await an answer.

### 5.3 Cytokine and Signaling Pathway Modulation

#### 5.3.1 Low‐Dose IL‐2

High‐dose IL‐2 favors inflammation by stimulating effector T cells. However, clinical trials confirmed that [254, 255, 256] a low dose of IL‐2 selectively expands regulatory T cells and maintains their suppressive functions, increases the Tr/Th ratio in mice [257] and SLE patients [258, 259], modulates Tfh cells and Th17 cells [256], expands NK cells [255], inhibits dsDNA antibodies, and improves renal conditions.

Complete remission without serious infection was achieved in patients with LN in the low‐dose IL‐2 group at week 12 in an RCT trial [255]. A low dose of alesleukin, a human recombinant interleukin‐2 product, was well tolerated in a phase II trial, LUPIL‐2, in active SLE without antidrug antibody generation [260]. Zhou et al. [261] reported that supplementation with low doses of IL‐2 improved SLE patients by alleviating Treg impairment resulting from prednisone. Several phase III trials will explore the combination of IL‐2 with other promising methods, such as human umbilical cord mesenchymal stromal cell (hUC‐MSCs) (NCT05631717 [198]), telitacicept (NCT05339217 [199]), and belimumab (NCT05262686 [200]).

Other IL‐2‐based drugs are currently in phase I or II trials. While CUG252, an IL‐2 mutein undergoing a phase I study in healthy volunteers (NCT05328557) [201], another mutated IL‐2 molecule, efavaleukin alfa, with higher IL‐2 Rα affinity, failed in a phase I trial (NCT03451422) [262], as all participants receiving AMG592 experienced treatment‐emergent adverse events (TEAEs). Rezpegaldesleukin (LY3471851), a PEGylated recombinant IL‐2, was confirmed in non‐central nervous system (CNS)‐ or non‐kidney‐involved active SLE patients at NCT04433585 [263]. Compared with the other IL‐2 groups or the placebo group, the intermediate dose of rezpegaldesleukin resulted in more patients achieving an SRI‐4 response at 24 weeks. Therefore, the results of IL‐2 analogs still await further study.

#### 5.3.2 Targeting the CD40–CD40 Ligand Signaling Axis

Although the CD40–CD40 ligand (CD40L) signaling axis is important for the abnormal development and maturation of T cells and B cells in SLE patients, many trials of anti‐CD40 antibodies, such as rupizumab (BG9588), ibanalumab (VAY736), and iscalimab (CFZ533) [264], have terminated or failed to meet primary endpoints [265, 266] because of adverse effects (AEs) or unsatisfactory therapeutic outcomes in SLE patients. Currently, a new CD40L inhibitor, dapirolizumab pegol, is under phase III multicountry clinical trial (NCT04976322 [202]) to evaluate its TEAEs and severe lupus flares in long‐term treatment with dapirolizumab pegol.

#### 5.3.3 Targeting Inducible Costimulators

ICOS, a molecule such as CD28, is involved in T‐cell activation when it engages with its ligand, ICOS‐L, on APCs. The monoclonal antibody prezalumab, which targets ICOS‐L, was safe for mild SEL in a phase II trial, but its clinical benefits need further careful confirmation [267].

#### 5.3.4 Targeting the JAK–STAT Pathway

The Janus kinase/signal transducer and activator of transcription protein (JAK/STAT) signaling pathway, with four kinases (JAK1‐3 and tyrosine kinase 2) and STATs significantly increased in SLE, is activated by various proinflammatory factors and is viewed as a potential therapeutic target.

Baricitinib is a potential therapeutic option for SLE because it selectively targets JAK 1/2. Baricitinib, at a dose of 4 mg in a phase II study, markedly alleviated the manifestations and indications of active SLE [268]. However, two phase III trials [269, 270] on baricitinib reported that it was ineffective. An ongoing phase III study of baricitinib in patients with LN (NCT05432531 [203]) is currently recruiting patients. More convincing evidence of barcitinib is awaiting.

Deucravacitinib is a new tyrosine kinase 2 (TYK2) that specifically targets the pseudokinase domain of TYK2 and inhibits IL‐6, IL‐12, IL‐23, and type I IFN signals downstream. NCT03252587 [271] reported that deucravacitinib resulted in higher SRI‐4 response rate in adult active SLE patients than placebo group. A randomized, double‐blind, placebo‐controlled phase III trial [204] on deucravacitinib with an estimated 490 enrollments is recruiting active SLE patients and planning to follow them for 156 weeks.

#### 5.3.5 Targeting the IFNα Receptor

Anifrolulmab is approved for moderate to severe SLE, as this monoclonal antibody antagonizes IFN‐alpha receptor and prevents signaling by type I IFNs. LN patients in a phase II study [272] were randomly assigned to receive the basic anifrolumab regimen, intensified regimen or placebo on standard therapy. While the primary endpoint was missing, a greater percentage of LN patients (45.5%) in the intensified group achieved a CRR than did those in the placebo group (31.1%) and sustained glucocorticoid reductions (55.6% and 33.3%, respectively). Further clinical trials are needed for the dose selection of intensified regimens in patients with LN.

Two multicenter phase III clinical trials are recruiting to examine anifrolumab in SLE patients and placebo for up to approximately 2 years. One of the two will focus on pediatric SLE patients (NCT05835310 [205]), whereas the other will enroll only adults (NCT05138133 [206]). Positive results are hoped for.

#### 5.3.6 Targeting IL‐17

The development of inflammation in organs involves Th17 cells producing cytokines such as IL‐17 [35]. Among the members of the IL‐17 family, IL‐17A is involved in the pathogenesis of SLE in both animal models and humans [71, 72]. Deletion of IL‐17 has even been shown to ameliorate the pathology of SLE [136]. Therefore, targeting IL‐17 is considered a promising therapeutic strategy for SLE. IL‐17 inhibitors are well established in psoriatic arthritis and spondyloarthritis [273, 274], yet clinical evidence supporting their efficacy in SLE remains limited. Secukinumab is a fully human IgG1κ targeting IL‐17A. While secukinumab is viewed as a promising therapeutic drug [275], cases have reported its beneficial use in LN patients as well as the unexpected occurrence of SLE in psoriatic arthritis patients [276, 277]. Only two phase II clinical trials (NCT04181762 [278] and NCT05232864 [279]) on LN have been terminated for unknown reasons, leaving the question of whether secukinumab is effective for SLE.

#### 5.3.7 Selective Bruton's Tyrosine Kinase Inhibitor

Katewa et al. [280] reported that a Bruton's tyrosine kinase (BTK) inhibitor could reduce gene expression signatures important for B‐cell differentiation in the spleen and ameliorate severe LN in NZB/W_F1 mice. Fenebrutini, an oral selective BTK inhibitor, reduced the BTK‐dependent plasmablast RNA signature, anti‐dsDNA autoantibodies, IgG/IgM levels and increased complement C4 by week 48 despite the unmet primary endpoint of SRI‐4 in a multicenter phase II trial [182]. Currently, no other trials on fenebrutinib in SLE have been conducted.

### 5.4 Novel Approaches

#### 5.4.1 Gut Microbiota Modulation

Gut microbiota alterations are associated with the onset and severity of SLE patients [281, 282]. The overall gut bacterial diversity decreased, including a reduction in probiotic bacteria and the proliferation of pathogenic bacteria. The techniques used to normalize the gut microbiota include dietary management and probiotics/prebiotics [283]. Among these methods, fecal microbiota transplantation (FMT) helps pave the way for the effective treatment of SLE [284, 285]. In mouse models, the gut microbiota from SLE mice significantly increased anti‐dsDNA antibodies and promoted an immune response in recipient germ‐free mice [285]. Interestingly, Huang et al. [284] first confirmed that FMT is safe and effective for individuals with SLE in a 12‐week pilot study with a single‐arm design. Twenty active SLE patients received oral capsules containing fecal microbiota from healthy individuals. No SAEs were reported after FMT. An SRI‐4 of 42.12% at week 12 was achieved as a primary endpoint. In addition, the SLEDAI‐2K score and the serum anti‐dsDNA antibody level decreased. SCFAs are elevated, and SCFA‐producing bacterial taxa in the gut are enriched. Inflammation‐related bacterial taxa were reduced, as were the levels of serum IL‐6 and the CD4+ memory/naive ratio. FMT capsules for active SLE patients will be further investigated in a nationwide multicenter RCT phase II clinical trial (ChiCTR2100052493 [207]), with 170 patients enrolled in China. This clinical trial is about to be unblinded.

#### 5.4.2 Human Umbilical Cord Mesenchymal Stromal Cells

MSCs can be isolated from bone marrow, the umbilical cord, adipose tissue, and other organs. Those derived from the umbilical cord are considered superior to those derived from other sources because of their noninvasive nature, lack of ethical concern, easy sampling and isolation, minimal immunogenicity, easy expansion in vitro, strong immunomodulatory function, and excellent proliferation potential. HUC‐MSC‐transplanted MRL/lpr lupus mice in the early stage or mild stage benefited, as the proliferation and differentiation of peripheral blood B cells were inhibited and kidney injury was alleviated [286].

A total of 10 clinical trials on the use of MSCs in SLE treatment have been registered on the Clinicaltrials.gov website, among which only three have reported results that are inconsistent. Two trials achieved promising results. In a single‐center single‐arm pilot trial [287], all 16 severe and refractory SLE patients completed UC‐MSC treatment as planned and were followed up at 1 and 3 months. Ten patients were followed up at 6 months, and two were followed up for more than 1 year. Disease activity indicators such as the SLIDAI, ANA, anti‐dsDNA, C3, C4, and renal ALB levels were significantly lower in all 16 patients without treatment‐related death or other AEs. A multicenter observation of 40 active SLE patients revealed that 32.5% (13 out of 40) and 27.5% (11 out of 40) achieved major or partial clinical response, respectively, at 12 months. Interestingly, disease relapse occurred in three patients at 9 months and four at 12 months. Thus, researchers suggest repeated MSC treatment after 6 months. However, a phase II trial [288] in 2017 revealed that more SLE patients experienced remission in the hUC‐MSC group than in the placebo group, and similar disease improvement was observed in both groups. More AEs occurred in the hUC‐MSC group: one patient experienced leucopenia, skin and lung infection, and another died of severe pneumonia. This study ceased afterward. Researchers optimized a phase I/II trial (NCT06485648 [289]) on MSC therapy for refractory LN in 2024 as a renal biopsy, and pathological confirmation will be applied for all enrolled patients to reduce bias.

Researchers are generally optimistic about the prospects of the use of MSCs in SLE. Previous failure may be due to physical contact between MSCs and immune cells in patients, as such contact may inhibit T effector cells and stimulate T regulatory cells in SLE [290]. A biocompatible microencapsulation technique was suggested to package MSCs and help isolate them from patient immune cells without disturbing soluble molecule secretion in SLE patients [290]. In a recent LN mouse study, pretreatment with dimethyloxallyl glycine was shown to enhance the therapeutic effect of hUC‐MSCs, probably via the TGF‐β/Smad pathway, by increasing their ability to reduce inflammation and counteract fibrosis [291]. It is hoped that strategies based on hUC‐MSCs will provide prospects for SLE patients.

#### 5.4.3 CAR‐T Therapy

Anti‐CD19 chimeric antigen T cell (CAR‐T) therapy has been shown to be effective in both prevention and treatment in preclinical SLE mouse models [292, 293], and similar satisfactory results have been reported in both adult and pediatric patients [183, 184, 185]. T cells derived from SLE patients were genetically modified using a lentiviral‐based vector that encodes an anti‐CD19 CAR. These modified T cells were then cultured, expanded, and infused back into patients who had already undergone lymphodepletion [183]. In 2022, all five refractory SLE patients receiving anti‐CD19 CAR‐T‐cell therapy achieved B‐cell depletion, decreased anti‐dsDNA antibodies, ameliorated clinical signs and renal involvement, and more importantly, achieved remission at 3 months post CAR‐T‐cell therapy, which even persisted through a median 8‐month follow‐up despite B‐cell recovery [183]. No AEs, such as infection or hemodynamic alterations, were reported [183]. In 2024, Müller et al. [10] reported no SLE disease activity during long‐term follow‐up of up to 29 months in all eight severe SLE patients who underwent CD19 CAR‐T‐cell therapy. In addition, anti‐dsDNA antibodies decreased and remained negative, the level of complement C3 returned to normal, and proteinuria resolved throughout the entire observation period. Interestingly, clinical trials typically exclude patients with CNS‐SLE. However, in a recent case, a CNS‐SLE patient was successfully treated with CD19 CAR‐T‐cell therapy [186], highlighting the potential for this treatment in patients with severe organ manifestations. More than 20 phase I or phase I/II clinical trials are enrolling SLE patients using anti‐CD19 CAR‐T‐cell therapies, such as NCT05765006 [208] and NCT06189157 [209], which may offer more convincing evidence of long‐term remission in larger populations in the future.

There are other exploratory allogeneic anti‐CD19 CAR‐immune cell strategies, such as anti‐CD19 CAR‐NK cell therapy (such as NCT06518668 [210] and NCT06421701 [211]), CD19/CD20 CAR‐T‐cell therapy (NCT06462144 [212] and NCT06567080 [213]), anti‐BCMA/CD19 CAR‐T‐cell therapy (NCT06428188 [214] and NCT06350110 [215]), anti‐CD19‐CD3E‐CAR‐T‐cell therapy (NCT06373081 [294]), and CD19‐CAR‐DNT‐cell therapy (NCT06340490 [216]). These studies are almost all phase I trials and have no results yet.

#### 5.4.4 Alternative Complement Inhibitors

With increasing research on the pathogenesis of SLE, the development of complement therapy as a novel treatment strategy has gradually attracted increasing attention. LNP023 is an oral medication that serves as an alternative complement inhibitor. It persistently reduced proteinuria without death, TEAEs, or bacterial infection for IgA nephropathy in a phase II study [295], whose role will be further tested in a phase III trial (NCT04578834 [296]). LNP023 was also tested in patients with lupus. A mouse study [187] revealed that the application of LNP023 in MRL/lpr mice ameliorated lupus‐like features; enhanced kidney function; and decreased serum anti‐dsDNA and anti‐ANA concentrations and complement deposition in the kidney. Renal pathological involvement was relieved, with reduced immune element deposition and inflammatory cell infiltration. A phase II multicenter RCT [217] will test LNP023 in active LN patients.

### 5.5 Clinical Trial Landscape

Clinical trials for SLE have yielded both success and failure. Success, such as the use of belimumab in phase III clinical trials, has demonstrated that targeting key components, such as B cells, holds great promise for SLE treatment. However, this approach is not always effective for patients. Importantly, numerous trials for SLE have failed. There are several reasons for these failures. First, off‐target effects play a role. In such cases, the therapy affects unintended molecular pathways, leading to compromised efficacy or severe side effects. Second, safety concerns such as infections caused by immunosuppression therapy cannot be ignored. Third, the mechanisms of SLE are diverse, with disease heterogeneity being a dominant characteristic. The predominant pathogenic mechanisms may differ among patients. These factors may jointly contribute to the challenges in clinical trials.

### 5.6 Challenges in Target Validation

The challenges in target validation for SLE are multifaceted. The first challenge stems from the complex pathogenesis of the disease, characterized by dysregulation of multiple immune components and intertwined signaling pathways. Such intricate biological nature obscures the identification of specific therapeutic targets closely related to disease onset and progression. Furthermore, the lack of specific, sensitive, and dynamic biomarkers interferes with the accurate identification and validation of targets. Moreover, the limited availability of biomarkers for early diagnosis and patient stratification presents another challenge. In addition, individual differences among SLE patients, shaped by genetics, the environment and lifestyle, lead to substantial variations in treatment responses, making it arduous to locate a one‐size‐fits‐all target. Notably, existing SLE animal models can only partially mimic human disease features and mechanisms, neutralizing the effectiveness of targets validated in animals in clinical trials. As SLE is a complex chronic disease, larger sample sizes and extended follow‐up are needed.

## 6 Conclusion and Prospects

Despite advances in understanding SLE pathogenesis, the currently known mechanisms still fail to fully explain its complex clinical phenotypes and pathology. Classical animal models also struggle to fully replicate clinical phenotypes, and their differences from human pathogenesis impede studies on disease origins and local immune microenvironments. The blurred boundaries between persistent disruption of immune tolerance and immune defense suggest that SLE onset is likely a chronic and insidious process.

In addition to systemic changes in lupus, the distortion of local immune microenvironments warrants greater attention from researchers. Studies on the kidney and skin have provided paradigms for epithelial‒immune cell crosstalk in SLE, expanding our understanding of the key cells involved in this disease. These studies underscore the significant role of the broad sense of innate immune responses in the pathogenesis of SLE. Moreover, as a multiorgan disease, neuropsychiatric lupus, as well as lupus‐related pulmonary and serosal involvement, may also exhibit unique inflammatory phenotypes and cellular interactions, which still require further investigation.

In terms of translational research, given the lack of effective therapies and the challenges in translational research, focusing on preclinical changes and identifying predictive biomarkers may become key strategies in future disease prevention and management. The ongoing exploration of novel therapeutic targets, including B‐cell and T‐cell modulations, holds promise for improving patient outcomes. Furthermore, strategies aimed at altering the gut microbiome or manipulating specific immune cell populations, such as regulatory T cells, are exciting potential strategies for therapeutic intervention. These approaches aim not only to control disease activity but also to address immune dysregulation, offering the potential for long‐term disease management. Considering the high patient heterogeneity, exploring environmental, genetic, and hormonal influences to achieve precise stratification and personalized treatment offers a promising approach for improving patient outcomes.

## Author Contributions

Conceptualization: J.T. and Q.L. Visualization: J.T. and H.Z. Funding acquisition: Q.L., J.T., W.L., and X.Y. Project administration: Q.L. and X.Y. Supervision: J.T. and Q.L. Writing—original draft: J.T. and H.Z. Writing—review and editing: J.T., H.Z., W.L., X.Y., and Q.L. All authors have read and approved the final manuscript.

## Ethics Statement

The authors have nothing to report.

## Conflicts of Interest

The authors declare no conflicts of interest.

## Supporting information



Supporting Information

## Data Availability

The authors have nothing to report.
