# Supplementary material for: New Mechanisms and Therapeutic Targets in Systemic Lupus Erythematosus
Source: MedComm (2020). 2025 Jun 9;6(6):e70246. doi: 10.1002/mco2.70246 (PMC12146670; doi:10.1002/mco2.70246)

New Mechanisms and Therapeutic Targets in Systemic Lupus Erythematosus

Authors: Jingru Tian[1,2,3,4†], Hang Zhou[1,2,3†], Wei Li [5], Xu Yao[1,4], and Qianjin Lu[1,2,3*]

Affiliations:

[1]Hospital for Skin Diseases, Institute of Dermatology, Chinese Academy of Medical Sciences and Peking Union Medical College, Nanjing, China.

[2]Key Laboratory of Basic and Translational Research on Immune-Mediated Skin Diseases, Chinese Academy of Medical Sciences, Nanjing, China

[3]Jiangsu Key Laboratory of Molecular Biology for Skin Diseases and STIs, Nanjing, China

[4]Department of Allergy and Rheumatology, Hospital for Skin Diseases, Institute of Dermatology, Chinese Academy of Medical Sciences and Peking Union Medical College, Nanjing, China

[5]Department of Dermatology, Huashan Hospital, Fudan University, Shanghai, China

* Correspondence should be addressed to:
Qianjin Lu, 12 Jiangwangmiao Street, Xuanwu, Nanjing, Jiangsu, China 210042.
Telephone: 025-85478999; Fax: 025-85414477;
Email: qianlu5860@pumcderm.cams.cn

† These authors contributed equally to this work

Figure S1. Compare the predicted number of SLE patients using our model with the actual number of SLE patients in prevalence studies.

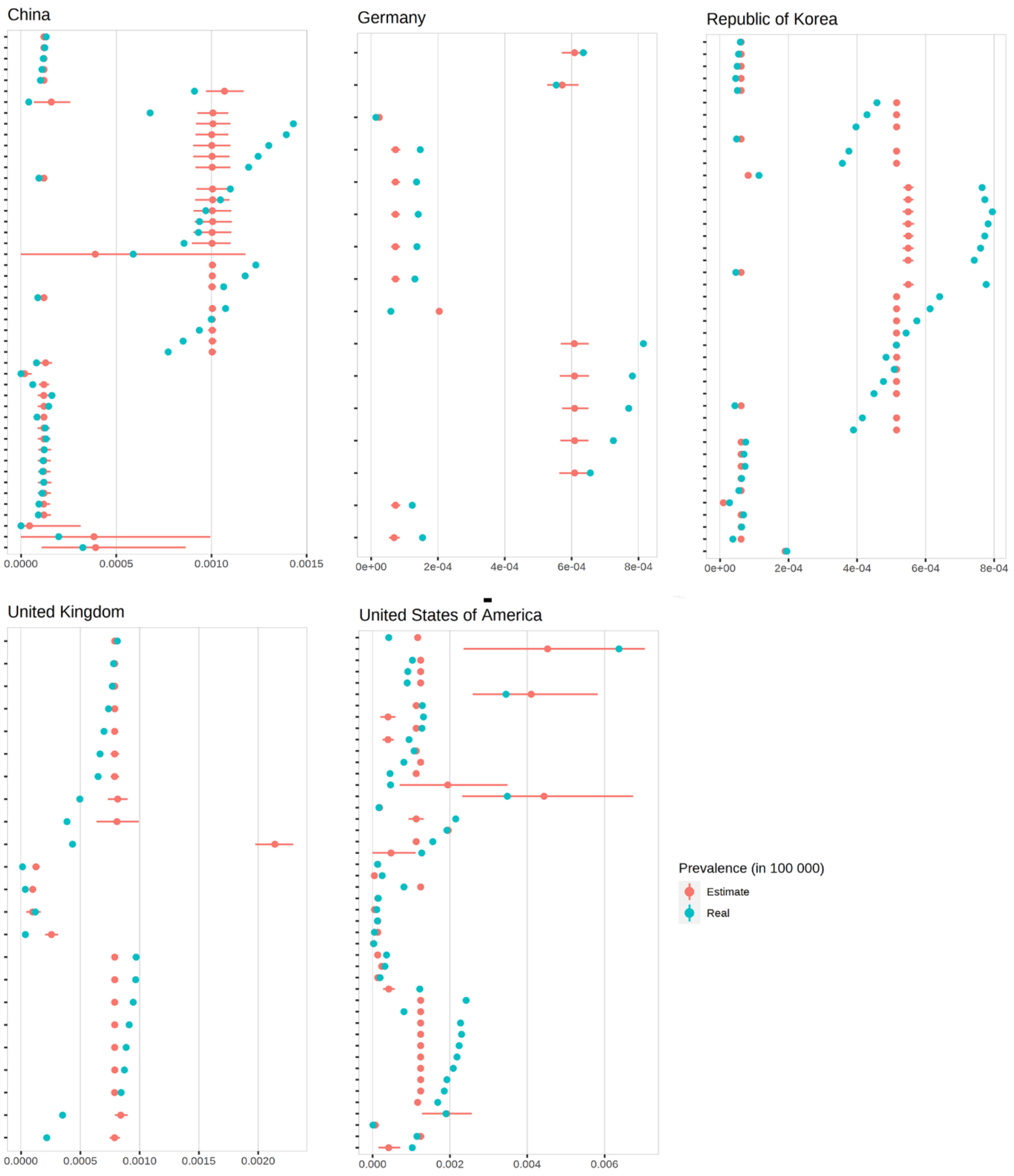

Figure S2. Compare the predicted number of SLE patients using our model with the actual number of SLE patients in incidence studies.

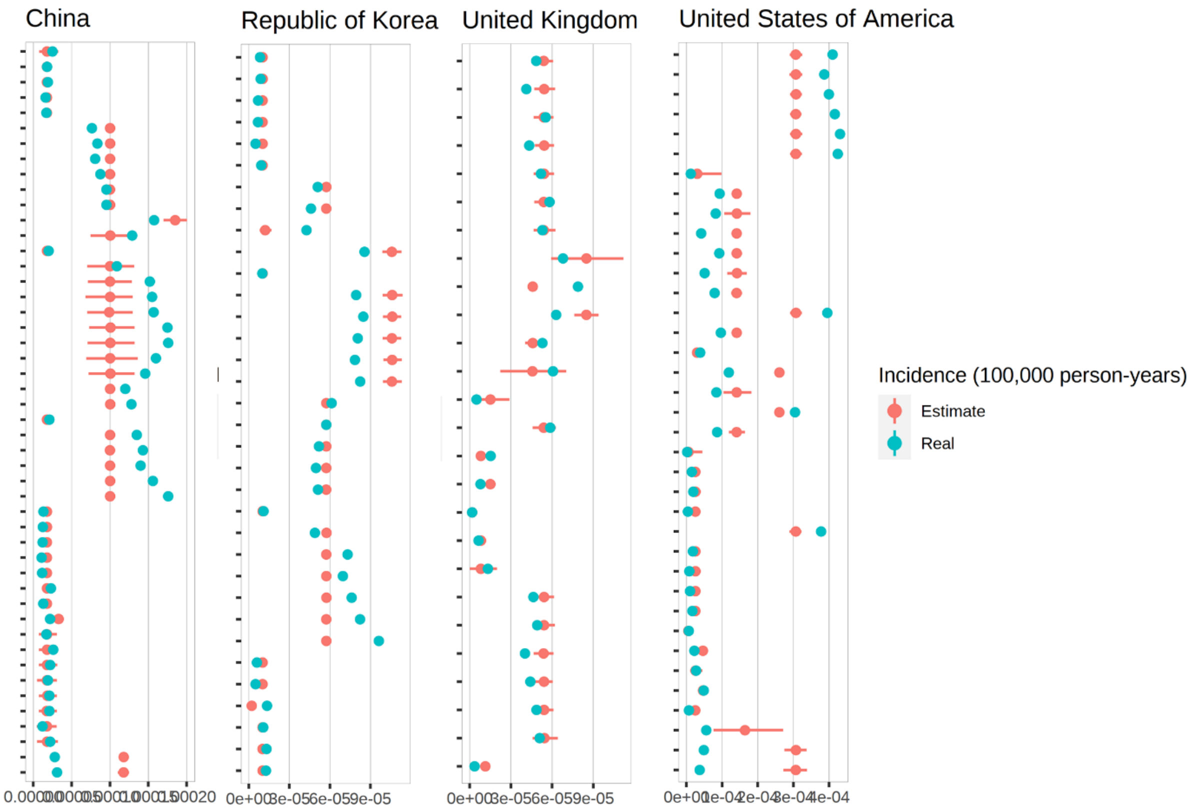

Supplement: Supplementary file 1 — Supporting Information [file MCO2-6-e70246-s001.pdf]
